# Successful harmonization in EpiBioS4Rx biomarker study on post-traumatic epilepsy paves the way towards powered preclinical multicenter studies

**DOI:** 10.1016/j.eplepsyres.2023.107263

**Published:** 2023-11-24

**Authors:** Xavier Ekolle Ndode-Ekane, Idrish Ali, Cesar E. Santana-Gomez, Pablo M. Casillas-Espinosa, Pedro Andrade, Gregory Smith, Tomi Paananen, Eppu Manninen, Riikka Immonen, Noora Puhakka, Robert Ciszek, Elina Hamäläinen, Rhys D. Brady, Juliana Silva, Emma Braine, Matthew R. Hudson, Glenn Yamakawa, Nigel C. Jones, Sandy R. Shultz, David Wright, Neil Harris, Olli Gröhn, Richard J. Staba, Terence J. O’Brien, Asla Pitkänen

**Affiliations:** a A.I. Virtanen Institute for Molecular Sciences, University of Eastern Finland, PO Box 1627, FI-70211 Kuopio, Finland; b Department of Neuroscience, Monash University, Australia; c Department of Neurology, Alfred Health, Australia; d Department of Medicine, The University of Melbourne, Australia; e Department of Neurology, David Geffen School of Medicine at UCLA, Los Angeles, CA, USA; f Department of Neurosurgery, David Geffen School of Medicine at UCLA, Los Angeles, CA, USA

**Keywords:** Common data element, Epileptogenesis, Lateral fluid-percussion injury, Magnetic, Resonance imaging, Plasma sampling, Traumatic brain injury, Videoelectroencephalogram

## Abstract

**Objective:**

Project 1 of the Preclinical Multicenter Epilepsy Bioinformatics Study for Antiepileptogenic Therapy (EpiBioS4Rx) consortium aims to identify preclinical biomarkers for antiepileptogenic therapies following traumatic brain injury (TBI). The international participating centers in Finland, Australia, and the United States have made a concerted effort to ensure protocol harmonization. Here, we evaluate the success of harmonization process by assessing the timing, coverage, and performance between the study sites.

**Method:**

We collected data on animal housing conditions, lateral fluid-percussion injury model production, postoperative care, mortality, post-TBI physiological monitoring, timing of blood sampling and quality, MR imaging timing and protocols, and duration of video-electroencephalography (EEG) follow-up using common data elements. Learning effect in harmonization was assessed by comparing procedural accuracy between the early and late stages of the project.

**Results:**

The animal housing conditions were comparable between the study sites but the postoperative care procedures varied. Impact pressure, duration of apnea, righting reflex, and acute mortality differed between the study sites (p < 0.001). The severity of TBI on D2 post TBI assessed using the composite neuroscore test was similar between the sites, but recovery of acute somato-motor deficits varied (p < 0.001). A total of 99% of rats included in the final cohort in UEF, 100% in Monash, and 79% in UCLA had blood samples taken at all time points. The timing of sampling differed on day (D)2 (p < 0.05) but not D9 (p > 0.05). Plasma quality was poor in 4% of the samples in UEF, 1% in Monash and 14% in UCLA. More than 97% of the final cohort were MR imaged at all timepoints in all study sites. The timing of imaging did not differ on D2 and D9 (p > 0.05), but varied at D30, 5 months, and ex vivo timepoints (p < 0.001). The percentage of rats that completed the monthly high-density video-EEG follow-up and the duration of video-EEG recording on the 7th post-injury month used for seizure detection for diagnosis of post-traumatic epilepsy differed between the sites (p < 0.001), yet the prevalence of PTE (UEF 21%, Monash 22%, UCLA 23%) was comparable between the sites (p > 0.05). A decrease in acute mortality and increase in plasma quality across time reflected a learning effect in the TBI production and blood sampling protocols.

**Significance:**

Our study is the first demonstration of the feasibility of protocol harmonization for performing powered preclinical multi-center trials for biomarker and therapy discovery of post-traumatic epilepsy.

## 1. Introduction

Post-traumatic epilepsy (PTE) comprises 10–20% of structural epilepsies (Herman, 2002). Despite about 20 favorable preclinical proof-of-concept trials and over 10 biomarker candidates, there are no treatments to combat epileptogenesis after traumatic brain injury (TBI) in clinic (Dulla and Pitkänen, 2021). To facilitate the diagnosis of epileptogenesis and the development of new antiepileptogenic treatments, statistically powered controlled pre-clinical studies are urgently needed (Galanopoulou et al., 2012; Simonato et al., 2014). Towards this goal, application of multi-center trial designs in preclinical research has been proposed (Balduini et al., 2016; Bath et al., 2009; Dirnagl et al., 2013; Lefer and Bolli, 2011; O’Brien et al., 2013). However, a successful multi-center study relies on systematic data collection, harmonization of experimental protocols and procedural rigor among the participating centers, which is still a rather uncharted territory.

The field was pioneered by the international research consortium for preclinical assessment of cardioprotective therapies (CEASAR) that demonstrated a successful implementation of standardized protocols among participating centers(Jones et al., 2015; Lefer and Bolli, 2011). In the field of neuroscience, the first preclinical multicenter randomized controlled trial (pRCT) tested the efficacy of anti-CD49d treatment for acute brain ischemia in two stroke models among six centers and demonstrated the feasibility and relevance of protocol harmonization (Llovera et al., 2015). Since then, Operation Brain Trauma Therapy network (OBTT) initiated an effort to conduct preclinical multi-center biomarker and therapy studies in TBI (Kochanek et al., 2018). More recently, methods optimization for preclinical stroke intervention studies have been reported by Italian Stroke Organization (ISO) Basic Science Network (Tettamanti et al., 2020) and the Stroke Preclinical Assessment Network (SPAN) investigators (https://spannetwork.org/; (Lyden et al., 2022)). These projects have demonstrated promising data, supporting the feasibility and benefits of conducting pre-clinical multi-center trials.

The Epilepsy Bioinformatics Study for Antiepileptogenic Therapy (EpiBioS4Rx) is a NINDS-funded Center-Without-Walls project, aiming to facilitate the development of antiepileptogenic therapies following traumatic brain injury (TBI) through identification of preclinical blood, electrophysiologic, and imaging biomarkers (https://epibios.loni.usc.edu/). The three preclinical study sites focusing on biomarker discovery in EpiBioS4Rx Project 1 are located in Finland (University of Eastern Finland), Australia (Monash University) and the United States (David Geffen School of Medicine at UCLA). For the first time, the EpiBioS4Rx Project 1 is implementing the use of common data elements (CDEs) in data collection, which were tailored for the needs of the study with the guidance of the work supported NINDS, the American Epilepsy Society and the International League Against Epilepsy (Harte-Hargrove et al., 2017; Pitkänen et al., 2019; Scharfman et al., 2018). However, it remains to be shown that the use of CDEs is doable and feasible in multi-center setup.

Previously, we published an *interim* analysis of the success of our procedural harmonization in the EpiBioS4Rx study between February 1, 2017 and April 30, 2018 (Pitkänen et al., 2019). The analysis included harmonization of the production of the animal model (Ndode-Ekane et al., 2019), blood sampling (Kamnaksh et al., 2019), magnetic resonance imaging (MRI) analysis (Immonen et al., 2019), EEG analysis (Casillas-Espinosa et al., 2019; Santana-Gomez et al., 2019) and metrics for analysis of success of harmonization (Ciszek et al., 2019). Now the experimental part of Project 1 has been completed and the animals have been phenotyped to diagnose PTE. Here our objective was (1) to assess inter-site success on timing and coverage of the experimental procedures specified in the study design, including TBI model production, blood sampling, MR imaging and video-EEG monitoring, (2) to assess the learning effect by comparing the performance between the early and late phases of the study, and finally, (3) to assess whether differences in the completeness of harmonization influenced the rate of epileptogenesis.

## 2. Materials and methods

**Fig. 1.**
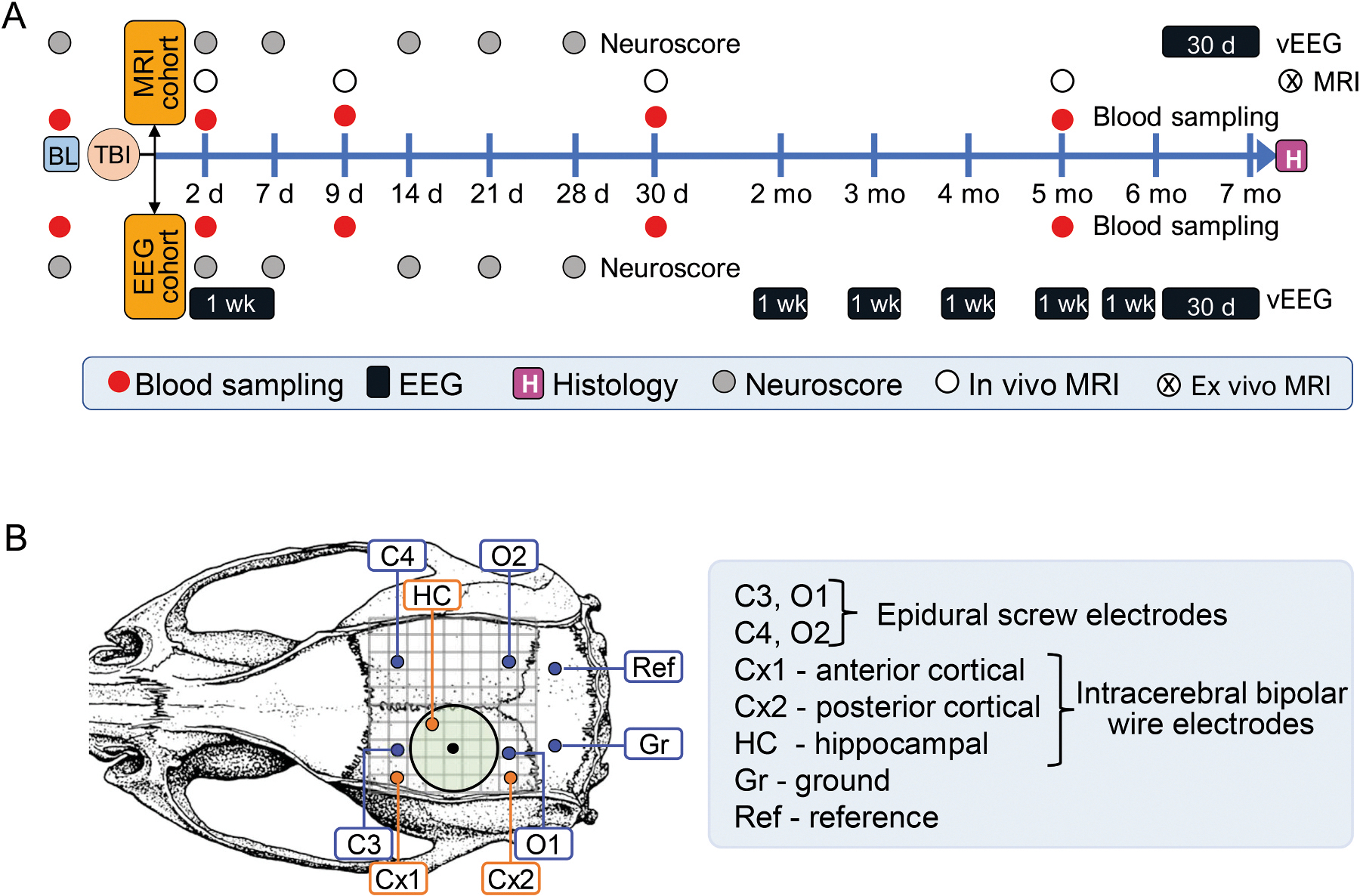
Study design and Electrode montage. (A) A schematic representation of the study design. EpiBioS4Rx Project 1 composed of two separate animal cohorts: the magnetic resonance imaging (MRI) and electroencephalography (EEG) cohorts. In both cohorts, the rats were randomized either into the sham-operated experimental control or TBI groups. Baseline blood sampling and the composite neuroscore test were performed prior to TBI or sham-operation. Thereafter, the blood was sampled on day (D) 2, D9, D30 and 5 months post-TBI. The composite neuroscore test was performed on D2, D7, D14, D21 and D28. In the MRI cohort, rats were MR imaged on day D2, D9, D30 and 5 months post-TBI. The order of test, for example on D2, was Neuroscore → blood sampling → MRI. Then, the rats were implanted with epidural and intracerebral electrodes for video-EEG recordings (see Methods). In the EEG cohort, electrodes were implanted right after TBI and rats were recorded monthly with a 1-wk video-EEG, starting right after the TBI or sham-operation till the 6th month. During the 7th month, both cohorts were continuously video-EEG monitored for 30 d to diagnose post-traumatic epilepsy. Finally, the rats were killed, and the brains were processed for ex vivo MRI and histology. **(B)** The electrode montage used in the study. Four epidural screw electrodes (C3, C4, O1 and O2), 3 bipolar intracerebral electrodes (Cx1, Cx2 and HC), ground (Gr) and reference (Ref). Abbreviations: BL, baseline; d, day; G, group; H, histology; mo, month; MRI, magnetic resonance imaging; TBI, traumatic brain injury; vEEG, video-electroencephalogram; wk, week.

EpiBioS4Rx is a National Institutes of Health -funded Centers-without-Walls Project that started in January 2017 (https://epibios.loni.usc.edu/). Project 1 is one of the five EpiBioS4Rx projects, focusing on discovery of preclinical blood, MRI and EEG biomarkers for epileptogenesis induced with lateral fluid-percussion injury (FPI) induced TBI in rats. The three study sites, including University of Eastern Finland (UEF, Kuopio, Finland), Monash University (Monash, Melbourne, Australia) and University of California in Los Angeles (UCLA, USA) followed the study design summarized in Fig. 1. The description of methodological protocols and the preliminary analysis of their implementation in preparation of the first 337 rats (143 UEF, 113 Monash and 81 UCLA) of a total of 524 were published earlier (Casillas-Espinosa et al., 2019; Ciszek et al., 2019; Immonen et al., 2019; Ndode-Ekane et al., 2019; Pitkänen et al., 2019; Santana-Gomez et al., 2019).

### 2.1. Ethics

#### UEF.

All animal procedures were approved by the Animal Ethics Committee of the Provincial Government of The Southern Finland and carried out in accordance with the guidelines of the European Community Council Directives 2010/63/EU.

#### Monash.

All animal procedures were approved by the Florey Animal Ethics Committee (ethics number 17–014 UM) at the University of Melbourne and by the Alfred Medical Research & Education Precinct Animal Ethics Committee (E/1799/2018/M) at the Monash University.

#### UCLA.

All animal procedures were approved by the University of California Los Angeles Institutional Animal Care and Use Committee (protocol 2000–153–61 A).

### 2.2. Power calculation of cohort size

Based on prior experiments in the participating groups, we anticipated the prevalence of epilepsy to be 25% after a 1-month video-EEG recording performed on the 7th post-TBI month (Kharatishvili et al., 2006; Shultz et al., 2013). Epilepsy was diagnosed according to the recommendation of the International League Against Epilepsy (Fisher et al., 2014). That is, the animal was considered to have PTE, if it had at least one unprovoked electrographic seizure or a handling-related seizure monitored by an experienced investigator on post-injury day (D) 8 or later. We anticipated a 30% acute (<48 h) post-impact mortality after induction of severe TBI and a 25% exclusion rate in the total cohort (e.g.*,* loss of electrode headset, weight decrease, skin infections, unexpected procedural or quality issues in blood, MRI and/or EEG data acquisition and/or analysis).

We expected to find a biomarker that will differentiate the TBI rats with epilepsy (TBI+) from those without epilepsy (TBI−) with AUC 0.700 (p < 0.05 as compared to 0.500; χ^2^ test, MedCalc software). Consequently, for each of the two specific aims (SA), we randomized a total of 189 rats into the TBI (n = 161) or sham-operated experimental control groups (n = 28). After considering the epileptogenesis rate and mortality/exclusions, we anticipated to have at least 21 TBI rats with epilepsy (TBI+), 63 TBI rats without epilepsy (TBI−) and 21 sham animals for the final biomarker analysis. The production of the MRI and EEG cohorts was equally divided between the three study sites (UEF, Monash and UCLA), each expected to generate (at least) 7 TBI+ , 21 TBI− and 7 sham-operated experimental controls for both the MRI and EEG cohorts (see below). Sham-operated experimental controls were generated to assess the direction of injury and/or epileptogenesis-induced changes in different biomarker candidates.

### 2.3. Data collection

By using the prior experience of Project 1 investigators (www.epitarget.eu) and the reports of the Working Group established by the American Epilepsy Society and the International League Against Epilepsy (ILAE) in collaboration with the National Institutes of Neurological Disorders and Stroke (NINDS) (https://www.ilae.org/research/resources) as well as the information available on the Common Data Elements developed for TBI research (Smith et al., 2015), the set of common data elements (CDEs) and the accompanying data dictionary applicable for the present study were generated. Additional CDEs were generated to complement the existing ones for the specific needs of this study.

The data were collected at each of the three study sites using excel sheets. For the analysis, data were combined and curated.

### 2.4. Experimental design

The animals were included into the study over a 3-year (y) period, the MRI cohort [Specific Aim 2 (SA2) of Project 1] being generated before the EEG cohort (SA1) (Fig. 1A).

In both cohorts, prior to TBI or sham-operation, a baseline tail vein blood sample was collected and sensory-motor performance was assessed using the composite neuroscore test. Thereafter, blood samples were collected on day D2 (48 h), D9, D30 and at 5 months (D0 is the injury day). The composite neuroscore was performed on D2, D7, D14, D21 and D28. In the MRI cohort, in vivo MRI was carried out on D2, D9, D30 and at 5 months after TBI or sham operation. In the EEG cohort, a high-density (HD) video-electroencephalography (video-EEG, except on UCLA) was started right after the induction of TBI or sham-operation, and continued for 1 week (wk), and thereafter, for 1 wk per month. During the 7th follow-up month, both cohorts were continuously video-EEG monitored for (at least) 30 d to diagnose the occurrence, frequency, duration and behavioral severity of unprovoked seizures. At the end, rats were killed, and the brains were processed for ex vivo MR imaging, and thereafter, for histology (Fig. 1A).

### 2.5. Animals

Adult male Sprague-Dawley rats were used at all study sites. In UEF and UCLA, the rats were purchased from the outside vendors (Table 1). In Monash, 75% were from the outside vendors (Table 1) and 25% (used in the MRI cohort) were produced by in-house breeding. The mean body weight of the rats at the start of the experiments (D0) in UEF was 354 ± 18 g (range 315 – 408 g, median 354 g), in Monash 349 ± 39 g (range 250 – 440 g, median 352) and in UCLA 336 ± 41 g (range 260 – 497 g, median 330). The body weight varied between the sites (p < 0.001, Kruskal-Wallis test).

### 2.6. Housing conditions

#### UEF.

All rats remained in quarantine for at least 1 wk upon arrival to the animal facility. Thereafter, rats were moved to single cages (48.5 cm × 28.5 cm×20 cm) till the end of the experiment. Seventy-eight % (143/184) of the rats were housed in ventilated cabinets in the holding room and 22% (41/184) in ventilated micro-isolation cages. The atmospheric and light conditions were similar in both housing conditions (temperature 22 ± 1 °C; humidity 50–60%; lights on from 07:00–19:00 h).

#### Monash.

The rats from the outside vendor remained in quarantine for 1 wk upon arrival. Thereafter, rats were moved to 2 rats per cage in the holding room. After TBI or sham-operation, rats were housed in single cages till the end of the follow-up. All rats were housed in ventilated cabinets with similar temperature, humidity, and lighting conditions as in UEF.

#### UCLA.

The rats remained in quarantine for 1 wk upon arrival to the animal facility. Thereafter, rats were moved (2 rats per cage) to the holding room and kept in ventilated cabinets. After TBI, rats were housed in single cages till the end of the study. The atmospheric and lighting condition included temperature 22 ± 1 °C, humidity 30–70% and lights on from 06:00–18:00 h.

### 2.7. Lateral fluid-percussion injury (FPI)

Lateral FPI was induced according to the protocol described by Kharatishvili et al. (2006) under isoflurane anesthesia (Ndode-Ekane et al., 2019; Shultz et al., 2013). Materials used at different study sites are summarized in Table 1. Briefly, rats were anesthetized with 5% isoflurane and then placed in a stereotactic frame. A midline insertion was made to expose the skull and a 5-mm craniotomy in diameter was made over the left convexity using a handheld trephine (UEF, UCLA) or a drill (Monash). After the surgery, the rat was disconnected from the stereotaxic frame. The duration of the surgery and anesthesia were recorded. The rat was connected to an FPI device after the toe pinch reflex had returned. Severe TBI (expected acute <48 h mortality 30%) (Pitkänen and McIntosh, 2006) was induced using a straight tip in the fluid-percussion device (Table 1).

The impact pressure on the brain was recorded. Following induction of TBI, the rat was monitored for (a) occurrence and duration of impact-induced seizure-like behavior, (b) duration of impact-induced apnea and (c) the latency it took the rat to fully right itself (righting time) on all four legs (righting reflex). Also, the duration of surgery and exposure to isoflurane anesthesia were recorded. All surgical procedures including TBI induction and electrode implantation was performed by the same person in UEF, three persons in Monash and UCLA.

### 2.8. Post-operative care

#### Post-operative analgesia.

***In UEF***, rats were treated with 0.05 mg/kg of buprenorphine right after the surgery. Treatment was repeated (once, 0.05 mg/kg) based on animal’s well-being, typically no longer than for 3 d. ***In Monash***, rats received buprenorphine at the beginning of the surgery, as instructed by the local animal ethics committee. ***In UCLA***, rats received 5 mg/kg, sc of flunixin meglumine (Flu-Nix) for post-operative analgesia (repeated every 12 h for 3 d),

#### Feeding.

***In UEF***, rats received powder pellet food (until they could eat on their own) and 0.9% NaCl (saline, s.c.; twice per day for 3 d). ***In Monash***, rats received soft food and saline with rodent milk in addition to saline (i.p.) as in UEF. ***In UCLA***, rats received chow supplemented with trimethoprim and sulfadiazine (TMS) pellet (ad libitum) and saline solution 0.9% (s.c.) (Ndode-Ekane et al., 2019).

At all sites, rats were monitored daily for changes in body weight, core temperature and other signs of disease or discomfort including general appearance (signs of pain), bowel and gestational function (stool nature), body conditioning score and any external bleeding.

### 2.9. Composite neuroscore

The neuroscore test was used to assess the magnitude of acute post-TBI somato-motor deficits as previously described (Ndode-Ekane et al., 2019; Nissinen et al., 2017). The test was performed prior to TBI or sham-operation, and then, on D2, D7, D14, D21 and D28 (Fig. 1A). Eight parameters were assessed: (i) left and right contraflexion, (ii) left and right hindlimb flexion, (iii) left and right lateral pulsion, and (iv) ability to stand on an inclined board in a vertical and horizontal (left and right) position. The maximum score is 28 (not impaired).

### 2.10. Blood sampling

Blood sampling and processing were performed using the previously described stepwise protocol (Kamnaksh et al., 2019; van Vliet et al., 2017). Details of the materials used at different study sites are described in Table 1. Briefly, 1 ml of blood was collected from the tail vain using a 23 G butterfly needle into two 500 μl K_2_EDTA tubes (tube A and tube B) before TBI (baseline) and on D2 (48 h), D9, D30 and at 5 months after TBI or sham-operation. The blood samples were centrifuged at 1300 g for 10 min at 4 °C. The quality of the plasma (degree of hemolysis) was assessed by measuring UV–vis absorbance of hemoglobin at 414 nm. Plasma sample with absorbance > 0.25 were considered of poor quality (Kamnaksh et al., 2019; van Vliet et al., 2017). The samples were aliquoted into bar-coded Eppendorf tubes, flash frozen and stored at − 80 °C.

The duration of exposure to anesthetics during the sampling procedure was recorded.

### 2.11. Magnetic resonance imaging

MRI was performed to assess the extent and evolution of structural pathology and connectivity after TBI. Rats in the MRI cohort (SA2) underwent in vivo MR imaging on D2, D9, D30 and at 5 months after TBI or sham-operation. The duration of each imaging session and exposure to anesthesia was recorded. At the end of the 7-months follow-up, rats in both cohorts were killed and the brains processed for ex vivo MRI as previously described (Immonen et al., 2019).

The in vivo MRI pulse sequences included multi-slice T2-weighted fast-spin echo (FSE) (edema, anatomy), 3D multi-echo gradient echo (MGRE) (susceptibility weighted imaging, SWI) with T2 * (anatomy), diffusion-weighted imaging (DWI), tractography and magnetization transfer (MT). Details of the MRI techniques and site-specific parameter adjustments have previously been published (Immonen et al., 2019).

The ex vivo MRI pulse sequences were the same as in vivo. However, minor adjustments were made to the acquisition parameters due to the faster relaxivity and lower temperature of fixed brain during imaging (Immonen et al., 2019).

### 2.12. Electrode implantation and video-EEG monitoring

Details of the video-EEG monitoring materials and parameters are summarized in Table 1.

#### Electrode implantation.

In the EEG cohort, electrodes were implanted right after LFPI induction; and in the MRI cohort at 6 months after the induction of TBI or sham-operation (Fig. 1A). The electrode montage included 4 epidural screw electrodes and 3 bipolar intracerebral electrodes (2 intracortical, 1 hippocampal) (Fig. 1B).

In both cohorts, the date of electrode implantation, duration of the surgery and duration of the exposure to isoflurane anesthesia were recorded.

#### Video-EEG recording.

The rats of the EEG cohort were connected to the video-EEG system right after electrode implantation and monitored for 1 wk. Thereafter, the 1-wk HD-EEG recording was repeated monthly over the next 6 months. During the 7th follow-up month, both cohorts were continuously video-EEG monitored for 30 d for diagnosis of PTE. Note that UCLA performed EEG without concomitant video monitoring.

### 2.13. Statistics

Data were analyzed using GraphPad Prism (version 9.3.1, GraphPad Software, LLC, USA) and IBM SPSS Statistics (version 27, IBM Corp., USA). The Shapiro–Wilk test was performed to test for normality. The Pearson chi square (χ^2^) test was used to test for site differences in the percentage of (1) acute mortality, (2) rats that completed all timepoints in each procedure (3) rats with electrodes implanted or reimplanted and (4) rats with epilepsy. The Kruskal-Wallis test followed by a post hoc analysis with Bonferroni correction for multiple tests was used to test for site differences in weight of rats at the start of study, impact pressure, apnea duration, righting time, blood sampling and MR imaging timepoints, plasma volume and quality, day of electrode implantation and duration of the 7th month video-EEG (phenotyping). The Friedman’s two-way analysis of variance was used to test differences in the quality of plasma between the sampling timepoints at different study sites. The mixed-effects model analysis was used to test for differences in recovery of acute somato-motor impairment (neuroscore), body weight and core temperature over the follow-up. The Mann-Whitney U test was used to test differences between the TBI+ and TBI− groups in (a) injury parameters (impact pressure, apnea, and righting time) and (b) duration of anesthesia exposure during surgery. Statistical significance was set at p < 0.05. All continuous data are presented as mean ± standard deviation (SD).

## 3. Results

### 3.1. Randomization

**Fig. 2.**
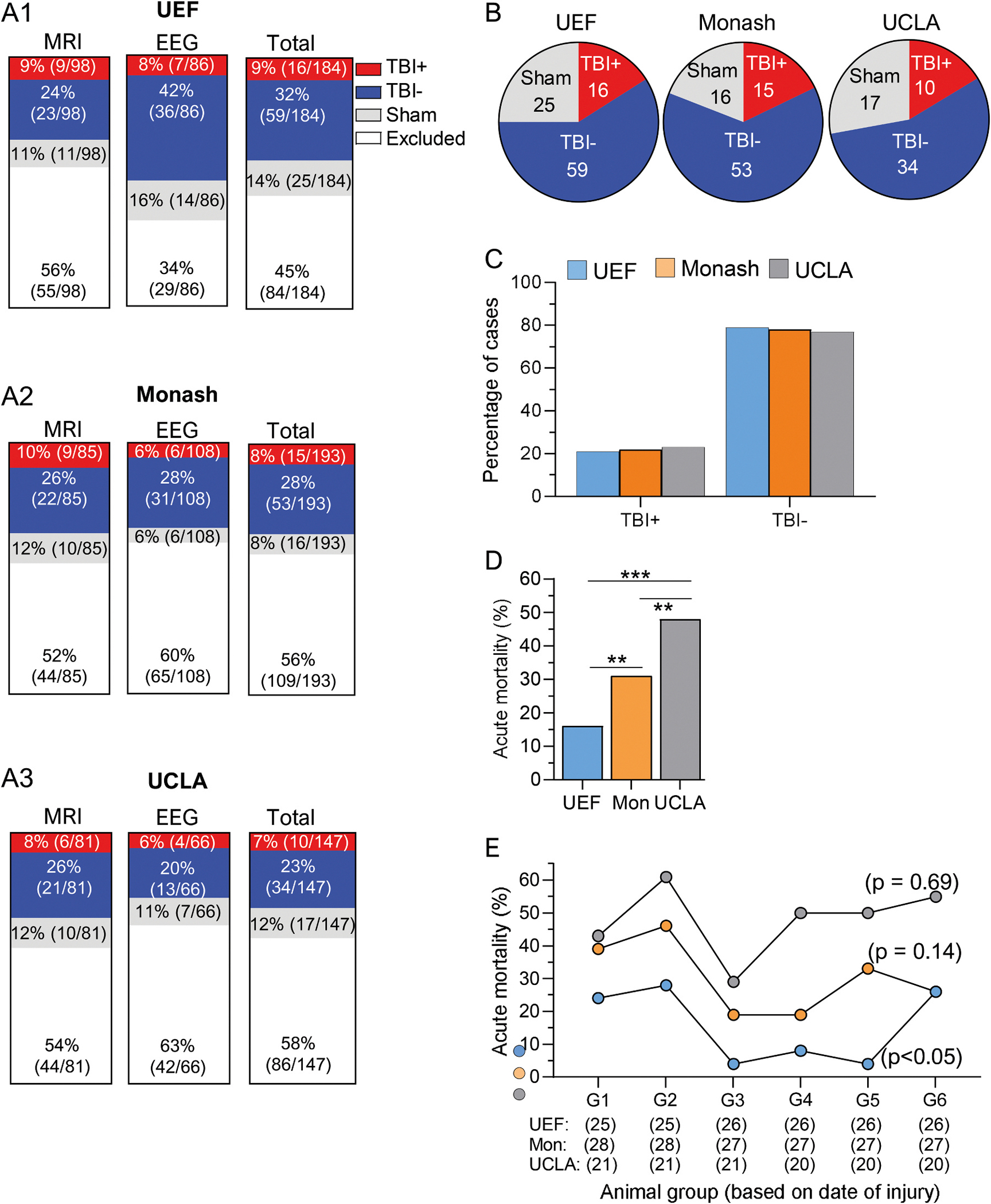
MRI and EEG cohorts, and acute mortality. (A1-A3) The graphs show the total number of animals recruited into the MRI and EEG cohorts at different study sites (UEF 184, Monash 193, UCLA 147). Also, the percentage of excluded and included cases in each sub-cohort and the total animal cohort are shown. (B) The final analysis cohort composed of 245 rats successfully phenotyped as sham (58), with epilepsy (TBI+, 41) or without epilepsy (TBI−, 146) during the 7th month video-EEG monitoring, and not excluded thereafter, due to brain abscess or other reasons (e.g., poor EEG quality). Note that each site was expected to produce 7 TBI+ , 21 TBI− and 7 sham-operated rats in both the MRI and EEG cohorts. (C) The percentage of rats with (TBI+) or without (TBI−) epilepsy in the final study cohort (MRI and EEG cohorts combined). There was no difference between the study sites. (D) The acute mortality (<48 h) differed between the study sites (p < 0.001, χ^2^ test). Statistical significances between the sites: * *, p < 0.01; * ** , p < 0.001 in panel F (χ^2^ test). (E) Acute mortality in UEF, Monash (Mon) and UCLA over the study progression (G1, the first animal group; G6, the last animal group). X-axis shows the number of animals in bracket. Except for UEF (p < 0.05), there was no difference between the groups in the other sites (Monash, p = 0.14; UCLA, p = 0.69; χ^2^ test). Abbreviations: TBI+ , rats with epilepsy; TBI−, rats without epilepsy; vEEG, video-electroencephalogram; χ^2^, chi square test.

A total of 524 rats were recruited into the study across all three study sites (264 into the MRI and 260 into the EEG cohort). Of these, 441 were randomized into the TBI group (221 to MRI and 220 to EEG cohort) and 83 into the sham group (42 to MRI and 41 to EEG cohort). Site-specific randomization is shown in Fig. 2A1–A3.

### 3.2. Number of animals included into the final analysis

#### Final analysis cohort.

Only the rats that had been successfully video-EEG phenotyped for epilepsy diagnosis at the end of the 7th month follow-up period entered the analysis cohort (Fig. 2A–B). This cohort was further polished from animals with poor quality EEG, brain abscesses detected in the ex vivo MRI and/or histology or death during electrode-re-implantation on the 7th month. Consequently, of the initial study cohort of 524 rats, 245 rats were included in the final analysis cohort (121 in MRI and 124 in EEG cohort) and 279 were excluded (143 from MRI and 136 from EEG cohort) (Fig. 2A and Table S1).

#### UEF.

After video-EEG phenotyping, 100 rats (43 in MRI and 57 in EEG cohort) were included in the biomarker analysis. Of these, 16/100 (16%) belonged to the TBI+ (9 in MRI and 7 in EEG cohort), 59/100 (59%) to the TBI− (23 in MRI and 36 in EEG cohort) and 25/100 (25%) to the sham-operated experimental control group (11 in MRI and 14 in EEG cohort) (Fig. 2B, C).

#### Monash.

Altogether 84 video-EEG phenotyped rats (41 in MRI and 43 in EEG cohort) were included in the final analysis. Of these 15/84 (18%) belonged to the TBI+ (9 in MRI and 6 in EEG cohort), 53/84 63% to the TBI− (22 in MRI and 31 in EEG cohort) and 16/84 (19%) to the sham group (10 in MRI and 6 in EEG cohort) (Fig. 2B, C).

#### UCLA.

Altogether 61 EEG-phenotyped rats (37 in MRI and 24 in EEG cohort) were included in the final analysis. Of these 10/61 (16%) belonged to the TBI+ (6 in MRI and 4 in EEG cohort), 34/61 (56%) to the TBI− (21 in MRI and 13 in EEG cohort) and 17/61 (28%) to the sham group (10 in MRI and 7 in EEG cohort) (Fig. 2B, C).

### 3.3. Exclusions

#### 3.3.1. Mortality

The acute and follow-up mortality at each study site was estimated from the total number of sham-operated (30 UEF, 29 Monash and 24 UCLA) or TBI rats (154 UEF, 164 Monash and 123 UCLA) recruited into the study (MRI and EEG cohorts combined).

##### 3.3.1.1. Acute mortality (<48 h).

The overall acute mortality at all study sites combined was 2% (2/83) in the sham group and 30% (133/441) in the TBI group. Acute mortality in the sham group was only reported by UCLA, being 8% (2/24). The percentage of acute mortality in the TBI group varied between the study sites, being 16% (24/154) in UEF, 31% (50/164) in Monash and 48% (59/123) in UCLA (p < 0.001, χ^2^ test) (Fig. 2D). No difference was found between the MRI and EEG cohorts (31% vs. 30%, p = 0.53).

###### Causes of acute mortality.

The death was related to TBI (mostly prolonged apnea).

###### Acute mortality over the study progression.

To verify whether the acute mortality in the rats randomized to the TBI group reduced over the course of the study along the improved experimental experience and training, TBI rats were divided into six sub-cohorts based on the chronological date of injury over the three years. In UEF, the acute mortality varied between the sub-cohorts (p < 0.05; Fig. 2E). The highest mortality was observed at the beginning in sub-cohort 2 (28%) and the lowest in sub-cohorts 3 and 5 (both 4%). In Monash (p = 0.14) and UCLA (p = 0.69), the mortality was comparable between the sub-cohorts (Fig. 2E). The acute mortality in UCLA was above 30% in all sub-cohorts over the time and was particularly high at the early phases of the project (60% in group 2; Fig. 2E).

##### 3.3.1.2. Follow-up mortality (≥ 48 h).

The overall follow-up mortality at all study sites combined was 1% (6/441) in TBI rats and 0% (0/83) in shams.

###### UEF.

The follow-up mortality was 1% (2/154). One TBI rat died unexpectedly at 3 months after TBI. Another TBI rat had a prolonged convulsive SE as the 1st seizure during the 7th month video-EEG recording, causing the death (the rat was included in the TBI+ group).

###### Monash.

The follow-up mortality was 1% (2/164). One TBI animal was found dead in cage and another TBI rat died during the electrode implantation surgery.

###### UCLA.

The follow-up mortality was 2% (2/123). One TBI rat died 2 wk after TBI and another from SE died 6 months after TBI.

#### 3.3.2. Non-mortality related exclusions

Non-mortality related causes of exclusions were evaluated from all animals recruited into the study (MRI and EEG cohorts combined; Table 2).

##### UEF.

Altogether 59 rats (43 in MRI and 16 in EEG cohort) were excluded for reasons other than mortality. Of these, 5/59 (9%) were sham (3 in MRI and 2 in EEG cohort) and 54/59 (91%) TBI rats (40 in MRI and 14 in EEG cohort).

##### Monash.

Altogether 16 rats (4 in MRI and 12 in EEG cohort) were excluded. Of these, 2/16 (13%) were sham (1 in MRI and 1 in EEG cohort) and 14/16 (87%) TBI rats (3 in MRI and 11 in EEG cohort).

##### UCLA.

A total of 10 rats (4 in MRI and 6 in EEG cohort) were excluded. Of these 3/10 (30%) were sham [2 in MRI and 1 in EEG cohort) and 7/10 (70%) TBI rats [2 in MRI and 5 in EEG cohort].

##### Causes of exclusions.

The most common reason for non-mortality-related exclusion reported by all sites was a broken dura after the impact (23% UEF, 6% Monash and 12% UCLA) (Table 2). Some exclusion criteria were unique to each experimental site. For example, in UEF 18% of the excluded cases were leftover (extra) TBI or sham-operated rats due to a lower-than-expected acute post-impact mortality. Twenty-% of exclusions related to a poor quality of the recorded EEG caused by non-functional pogo-pins in the electrode headset. In Monash, 24% of exclusions were due to a poor-quality EEG. In UCLA, 8% of the exclusions related to animal death during reimplantation of the EEG headset (Table 2).

### 3.4. TBI induction -related parameters

Next, we assessed inter-site variability in impact pressure, duration of post-impact apnea, righting reflex time and acute somato-motor deficits (neuroscore) in all cases recruited into the study (MRI and EEG cohorts combined).

#### Post-impact seizure-like behavior.

Following TBI, seizure-like behavior including rotation of the lower torso, tail rotation and hind or forelimb jerks was observed in 35% (26/76) of the final included TBI cohort in UEF and 4% (3/68) in Monash. UCLA did not record the acute post-impact seizure-like behavior.

#### Impact pressure.

**Fig. 3.**
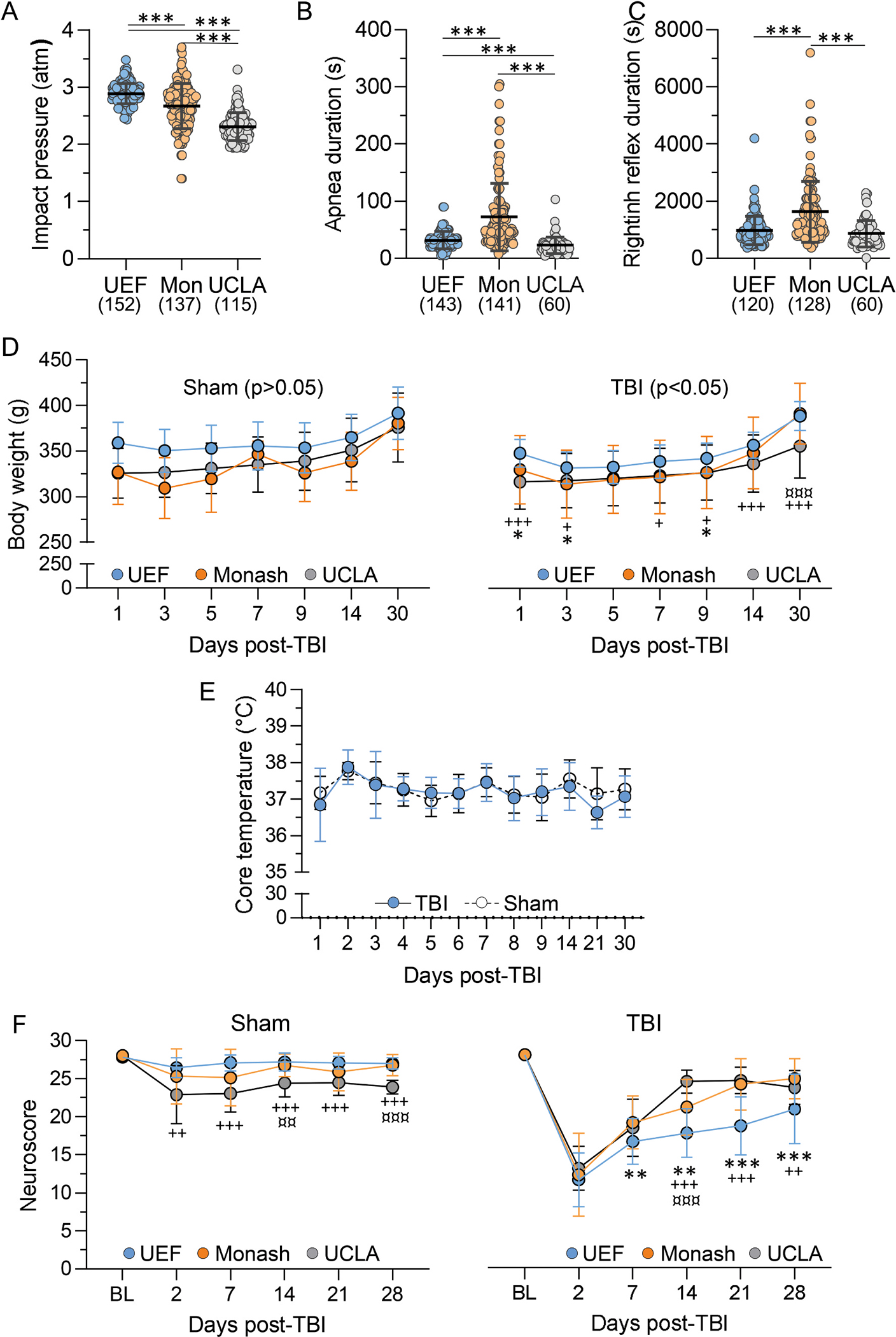
Parameters related to induction of TBI all animals recruited and assessment of acute somato-motor deficits in animals included in the final cohort. (A) The mean impact pressure differed between the sites (p < 0.001), being the lowest in UCLA. X-axis shows the number of animals in bracket. (B) The mean post-impact apnea duration also varied between the study sites (p < 0.001). The greatest inter-animal variability was observed in Monash. (C) The average time for a rat to self-right after TBI (righting reflex duration) differed between the sites (p < 0.001). The average time was longest in Monash with one rat taking more than 116 min to right itself. (D) The body weight assessment was used as an indirect measure of recovery after TBI. There was no difference in the body weight progression of sham rats between the sites. However, the body weight progression after TBI varied between the sites. (E) The core temperature assessment was also used as a criteria to evaluate the animals recovery and wellbeing after TBI. The assessment was performed only in UEF site. There was no difference in the core temperature progression between sham and TBI rats following injury. (F) The composite neuroscore test was used to assess acute post-impact somato-motor deficit. In the sham group, neuroscore differed between the sites (p < 0.001, mixed-effects model analysis), being the lowest in UCLA. In the TBI group, the neuroscore differed differ between the sites (p < 0.001, mixed-effects model analysis), being lower in UEF than in Monash or UCLA till D28. On D2, the neuroscore range was 7 – 17, suggesting moderate to severe injury. Note a drop in neuroscore on D2 at all sites. Data are presented as mean ± SD. Each circle presents the average at each timepoint. Statistical significances: * *p < 001, * **p < 0.001 UEF as compared to Monash; ++p < 0.01, +++p < 0.001 UEF as compared to UCLA; and ¤¤p < 0.01, ¤¤¤p < 0.001 Monash as compared to UCLA (Šídáks multiple comparisons test). Abbreviations: BL, baseline; Mon, Monash.

The mean impact pressure varied between the sites (p < 0.001) (Fig. 3A). In UEF, the impact pressure used (2.9 ± 0.18 atm, range: 2.4 – 3.5 atm, n = 152) was higher than that in Monash (2.7 ± 0.39 atm, range: 1.4 – 3.7 atm, n = 137) (p < 0.001) or in UCLA (2.3 ± 0.24 atm, range: 1.9 – 3.3 atm, n = 115) (p < 0.001) (Fig. 3A). In Monash, the impact pressure was higher than that in UCLA (p < 0.001).

#### Post-impact apnea.

The mean post-impact apnea varied between the sites (p < 0.001) (Fig. 3B). In UEF, the apnea duration (32 ± 15 s, range: 5 – 90 s, n = 143) was shorter than that in Monash (72 ± 59 s, range: 8 – 305 s, n = 141) (p < 0.001). Also, in UCLA, the apnea duration (23 ± 15 s, range: 4 – 103 s, n = 60) was shorter than that in Monash (p < 0.001) and also shorter than that in UEF(p < 0.001) (Fig. 3B).

#### Righting reflex.

The time it took for a rat to self-right (righting reflex duration) after the fluid-percussion -induced TBI varied between the sites (p < 0.001) (Fig. 3C). In UEF, the righting time (978 ± 489 s, range: 360 – 4200 s, n = 120) was shorter than that in Monash (1632 ± 1071 s, range: 360 – 7200 s, n = 128) (p < 0.001). Also in UCLA, the righting time (865 ± 464 s, range: 14 – 2280 s, n = 60) was shorter than that in Monash (p < 0.001) (Fig. 3C). In UEF and UCLA, the righting times were comparable (p = 1.00) (Fig. 3C).

### 3.5. Animal physiology

The analysis of body weight and core temperature focused on the cases included into the follow-up only as the data was most complete in these cases (MRI and EEG cohorts combined when data available on both cohorts).

#### 3.5.1. Body weight

In UEF body weight was assessed only in the MRI cohort and not in the EEG cohort to avoid destabilizing the electrode headset. In Monash and UCLA, body weight was assessed in both cohorts.

In UEF the body weight was assessed at baseline (BL) (prior to TBI, day 0) and then on D1, D2, D3, D4, D5, D6, D7, D8, D9, D14 and D30 after TBI. At Monash, rats were weighted at BL and then every day until animals recovered their acute unprovoked activity, coat condition, grimace, and weight loss. Subsequently, rats were weighted weekly. In UCLA animals were weighted at BL, D1, D3, D5, D7, D9, D14 and D30 post-TBI.

For the completeness of the dataset, site differences in post-TBI body weight progression were assessed using the UCLA timepoints. The body weight progression varied between sham and TBI rats in UEF (F_(1, 41)_ = 4.3, p < 0.05), but not in Monash (p = 0.78) or in UCLA (p = 0.16). There was no difference in the body weight progression of sham-operated rats between the sites (p = 0.06) (Fig. 3D). However, in TBI rats we found a difference in body weight over the follow-up (F_(2, 128)_ = 4.6, p = 0.012) (Fig. 3D).

#### 3.5.2. Core body temperature

Only UEF measured the core temperature. Measurements were done at the same timepoints as the body weight.

The baseline core temperature was comparable between the sham and TBI groups (p = 0.53). Similarly, the core temperature after TBI did not differ between the sham and TBI groups at any follow-up timepoint (p > 0.05) (Fig. 3E).

### 3.6. Post-impact somato-motor deficit

#### 3.6.1. Animal cohort

The composite neuroscore was used to assess the severity and recovery of the acute somato-motor deficit.(McIntosh et al., 1989) In UEF and Monash, the composite neuroscore test was not performed in the EEG cohort to avoid destabilizing the electrode headset that was implanted right after the TBI induction. In UCLA, the test was performed for both the MRI and EEG cohorts. Pooled data from all the sites showed that 189/524 (36%) of all rats initially recruited into the study (137 included in the final analysis cohort, 52 excluded) completed the neuroscore test.

##### Percentage of rats completing the test.

In the MRI cohort, 150/264 of the rats (57%; 112/150 included in the final analysis cohort, 38/150 excluded) completed the test. Overall, the percentage of cases in the final MRI analysis cohort that completed the neuroscore testing differed between the three study sites (see below; UEF 98% completed the test, Monash 81%, UCLA 100%, p < 0.01, χ^2^ test). In the EEG cohort (data from UCLA only), 39/260 of the rats (15%; 25/39 included in the final analysis cohort, 14/39 excluded) completed the composite neuroscore test.

##### UEF.

In the MRI cohort, 68/98 of the rats (69%; 42/68 included in the final analysis cohort, 26/68 excluded) completed all time points in the neuroscore test. Of the final included cohort, 42/43 (98%) completed the test. One rat included in the final included cohort did not complete the test.

##### Monash.

In the MRI cohort, 39/85 of the rats (46%; 33/39 included in the final analysis cohort, 6/39 excluded) completed all testing time points. Of the final included cohort, 33/41 (81%) completed all time points (p < 0.05 as compared to UEF, χ^2^ test). Eight rats included did not complete the test.

##### UCLA.

In the MRI cohort, 43/81 (53%; 37 included in the final analysis cohort, 6 excluded) completed the testing. Of the included cases, 37/37 (100%) completed all time points (p = 0.35 as compared to UEF, p < 0.01 as compared to Monash, χ^2^ test).

In the UCLA EEG cohort, 32/66 (49%; 24 included in the final EEG analysis cohort, 8 excluded) completed all timepoints. Of the included cases, 24/24 (100%) completed all neuroscore testing timepoints.

#### 3.6.2. Variability in the composite neuroscore between the sites

In UCLA, the test was performed in both the MRI and EEG cohorts. As the neuroscore in the injured (as well as in the sham-operated rats) was comparable between the MRI and EEG cohorts (p = 0.24), the UCLA data from both cohorts was pooled for further analysis.

##### Sham.

The neuroscore differed between the sites by testing day (F_(2, 66)_ = 10.87, p < 0.001) and the study site (F_(2, 35)_ = 31.05, p < 0.001). We also found an interaction between the study site and the testing day (F_(10, 170)_ = 2.28, p < 0.05) (Fig. 3F).

##### TBI.

The neuroscore differed by the testing day (F_(4, 394)_ = 408, p < 0.001) and the study site (F_(2, 107)_ = 34, p < 0.001). We also found interaction between the study site and the testing day (F_(10, 526)_ = 12, p < 0.001) (Fig. 3F).

##### *Sham* vs *TBI.*

***In UEF***, the groups differed by testing day (F_(4, 157)_ = 47, p < 0.001) and treatment (sham or TBI) (F_(1, 41)_ = 148, p < 0.001) with an interaction between the group and the testing day (F_(5, 205)_ = 35 p < 0.001). ***In Monash***, the groups also differed by the testing day (F_(3, 94)_ = 33, p < 0.001) and the treatment (F(1, 39) = 46, p < 0.001) with an interaction between the group and testing day (F_(5, 181)_ = 18, p < 0.001). ***In UCLA***, the groups differed by the testing day (F(3, 178) = 118, p < 0.001) and treatment (F_(1, 62)_ = 73, p < 0.001). The combined data further confirmed that groups differed by the testing day (F_(4, 539)_ = 152, p < 0.001) and the treatment (F_(1, 146)_ = 149, p < 0.001). there was also an interaction between the group and the testing day (F_(5, 716)_ = 68, p < 0.001).

### 3.7. Post-TBI blood sampling

#### 3.7.1. Number of animals sampled

The combined data from all three study sites show that 285/524 (54%) of all rats recruited (231 included, 54 excluded from the final analysis) were sampled at all 5 timepoints. 14 rats (out of a total of 245) included in the final study cohort were not sampled at all time points (Table 3).

##### UEF.

Altogether, 130/184 (71%) of the rats (99 included, 31 excluded from the final analysis) were sampled at each timepoint, including rats in both the MRI and EEG cohorts. Thus, out of a total of 100 cases included in the final analysis cohort, only 1 rat (died 3 months post-TBI) did not complete all time points (Table 3).

##### Monash.

Altogether, 103/193 (53%) of the rats (84 included, 19 excluded from the final analysis) were sampled at each timepoint, including rats in both the MRI and EEG cohorts. The included cases constituted 100% (84/84) of the final Monash analysis cohort (p = 0.19 as compared to UEF) (Table 3).

##### UCLA.

Altogether, 52/147 (35%) of the rats (48 included, 4 excluded from the final analysis) were sampled at each timepoint, including rats in both the MRI and EEG cohorts (Table 3). The included cases constituted 79% (48/61) of the final UCLA analysis cohort (p < 0.001 and p < 0.001 as compared to UEF and Monash respectively, χ^2^ test).

#### 3.7.2. Sampling timepoints

**Fig. 4.**
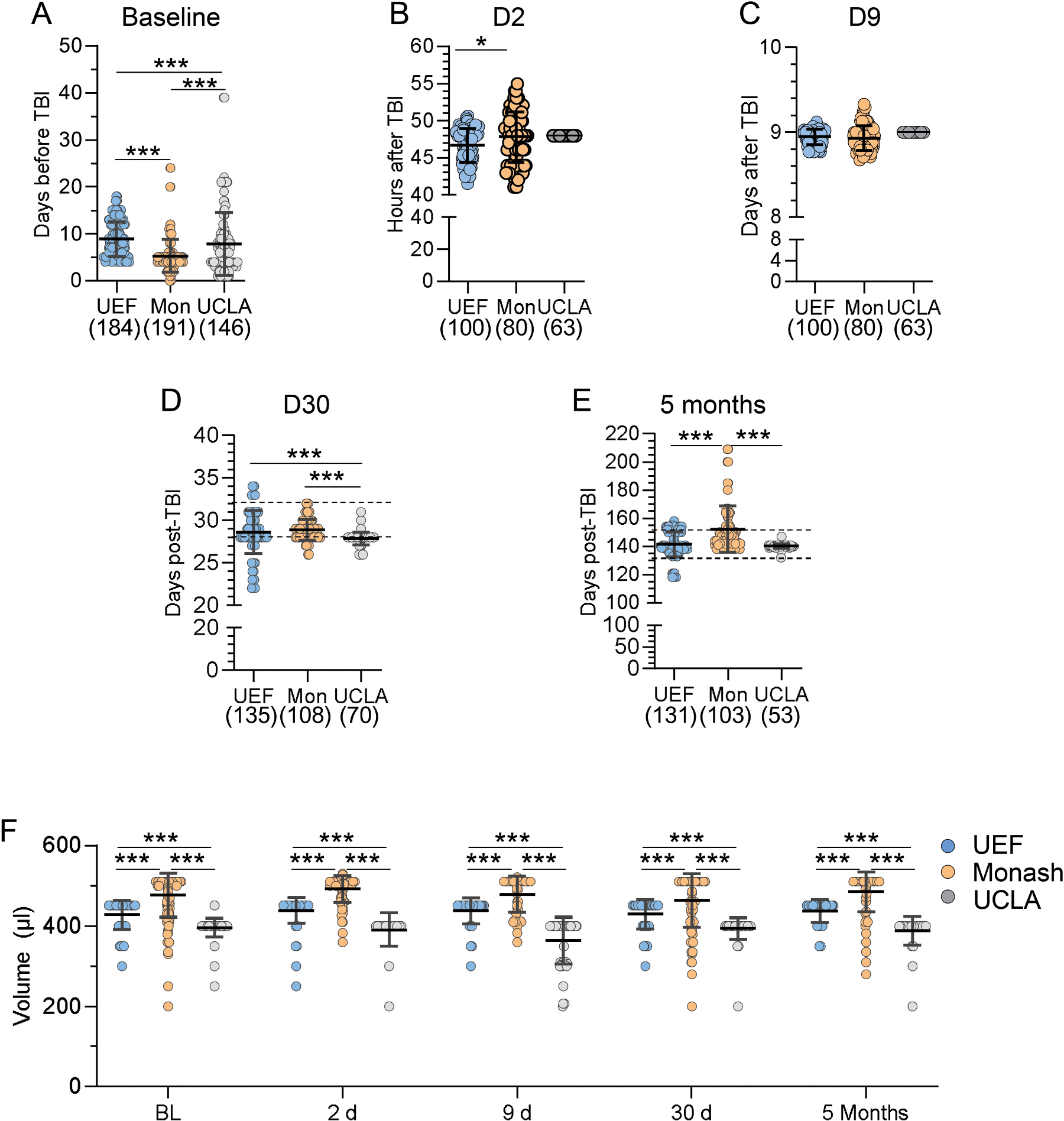
Timing of blood sampling and plasma volume. (A-E) The time delay from sham-operation or TBI to tail vein blood sampling differed between the sites on D2, D9, D30 and at 5 months. D2 and D9 were chosen for biomarker analysis, thus, to show the accuracy of timing we represent only the data of the final included cohort. The dashed lines indicate the pre-determined allowed deviation ( ± 2 d on D30 and ± 7 d at 5 months). Note that in UCLA, 1 rat was sampled at baseline more than a month before TBI. Moreover, the exact time of sampling on D2 and D9 was not recorded, thus, sampling timing could not be compared with the other sites. X-axis shows the number of animals in bracket. (F) The mean plasma volume in the EDTA tubes A and B varied between the sites, with the highest volume in Monash at all time points. Data are presented as mean ± SD. Each circle presents one animal. ***Statistical significances***: *p < 0.05, * *p < 0.01, * **p < 0.001 (Mann-Whitney test).

All rats were sampled on D2 and D9 post TBI. However, since the deviations in timing of the D2 (48 h) sampling is the most sensitive for affecting the biomarker analysis, we next analyzed the accuracy in timing of the D2 (48 h) blood sampling. The time from TBI to sampling was shorter in UEF (46.6 ± 2.3 h, range 41.5 – 50.6 h) than in Monash (47.8 ± 3.4 h, range: 41.0 – 54.9 h) (p < 0.05) (Fig. 4B). **On D9**, the accuracy of timing was comparable in UEF and Monash (p = 0.15) (Fig. 4C; data on the final analysis cohort). UCLA did not record the time of sampling on D2 and D9. On D30, the sampling was anticipated to occur at 30 d ± 2 d post-TBI. Data analysis revealed a site difference on D30 sampling (p < 0.001) (Fig. 4D). In UEF, 18% (24/135) of the D30 samples were collected <D28 and 8% (11/135) >D32. In Monash, 7% (7/108) were sampled <D28 and 0% >D32. In UCLA, 13% (9/70) were sampled <D28 and none >D32 (Fig. 4D). At 5 months, the sampling was anticipated to occur at 5 months ± 7 d post-TBI. Also, at this time point, we found a site difference in timing of sampling (p < 0.001) (Fig. 4E). In UEF, 35% (46/131) of the rats were sampled outside the anticipated time window, 52% (55/103) in Monash and 2% (1/53) in UCLA (Fig. 4E).

#### 3.7.3. Plasma volume

We aimed to separate plasma from 1.0 ml of blood withdrawn (divided into tubes A and B, 500 μl each) collected from every rat at each time point. The mean plasma volume (tube A and tube B combined) differed between the sites at all timepoints (p < 0.001) (Fig. 4F). At each timepoint, Monash had the highest and UCLA the lowest average plasma volume (Fig. 4F).

#### 3.7.4. Plasma quality

**Fig. 5.**
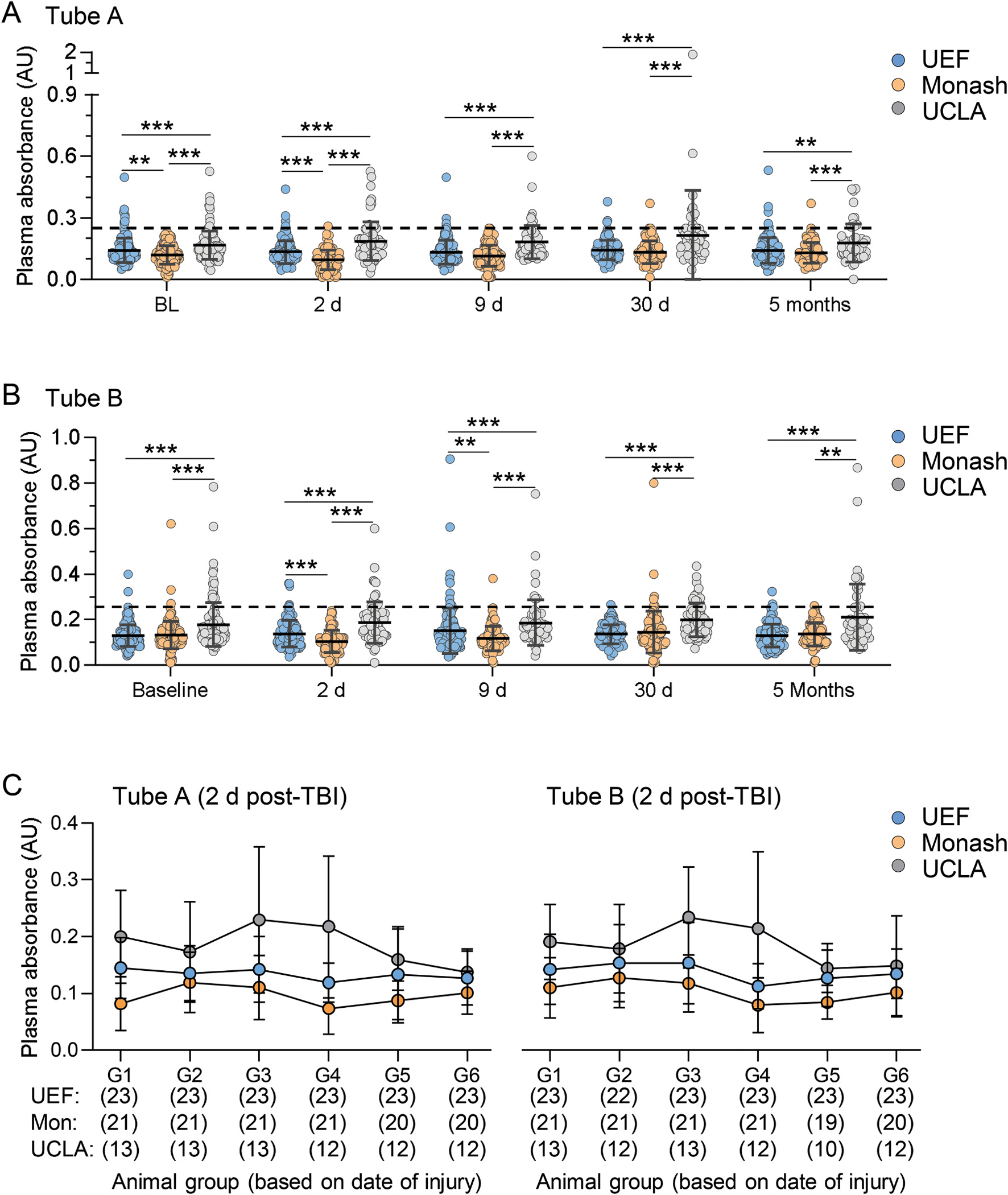
Plasma quality and intra-site sampling variability. (A) The mean plasma absorbance at 414 nm (indicator of hemolysis) in tube A. Despite the inter-site differences in plasma absorbance, the average absorbance was < 0.25 at all timepoints, which was the upper threshold for good quality plasma samples. Only a few samples (4% in UEF, 1% in Monash, 14% in UCLA) were discarded due to poor plasma quality. (B) The mean plasma absorbance at 414 nm in tube B also varied between the sites. Like in tube A, the mean absorbance was < 0.25 at all sampling timepoints. (C) The intra-site variability in the D2 plasma quality across the follow-up time. Rats were divided into 6 chronological groups based on the date of injury. In UEF and UCLA, the mean plasma absorbance did not vary between the sampling time points (Tube A: UEF, p = 0.29; UCLA, p = 0.13 and tube B: UEF, p = 0.16; UCLA, p = 0.061). In Monash, however, the mean plasma absorbance varied between the timepoints in tube A (p < 0.05) but not tube B (p = 0.12) with elevated values in both tubes observed early on (G2) but later decreases at as the study progressed (G4). X-axis shows the number of animals in bracket. Data are presented as mean ± SD. Each circle presents one animal. Statistical significances: *p < 0.05, * *p < 0.01, * **p < 0.001 (Mann-Whitney test).

The mean plasma absorbance at 414 nm (A414) differed between the sites at each sampling time point both in tube A (plasma closer to the surface, p < 0.001) and in tube B (plasma closer to the cellular pellet, p < 0.001) (Fig. 5A–B). Pooled data from all sites show that the mean plasma absorbance also differed between the time points both in tube A (p < 0.001) and tube B (p < 0.01).

The mean A414 values were within the acceptance limit (<0.25) at all time points at all study sites(Kamnaksh et al., 2019; van Vliet et al., 2017) (Fig. 5A–B). However, analysis (tube A and tube B combined) revealed that in 4% (52/1452) of the UEF samples, in 1% (15/1258) of Monash samples and in 14% (111/825) of UCLA samples the A414 was > 0.25 (p < 0.001, as compared between the sites).

In UEF and UCLA, the mean plasma absorbance did not vary between the timepoints in tube A (UEF, p = 0.13; UCLA, p = 0.063) or in tube B (UEF = 0.31; UCLA = 0.085) (Fig. 5A–B).

In Monash, the quality of the plasma varied between the timepoints both in tube A (p < 0.01) and tube B (p < 0.01) (Fig. 5A–B). In tube A, the lowest mean absorbance value was observed on D2 (0.09 ± 0.05) and the highest on D30 post-TBI (0.13 ± 0.06). In tube B, the lowest mean absorbance value was observed on D2 (0.10 ± 0.05) and the highest on D30 post-TBI (0.14 ± 0.09) (Fig. 5A–B).

##### Variability of plasma quality over the project progression.

To verify the quality of plasma along the project progression and prepare a “sampling learning curve”, the rats at each study site were divided into 6 sub-groups based on the chronological date on injury (group 1 first, group 6 last). The assessment included the D2 sampling time point only, since it was the most sensitive to variability in sample quality due to (1) difficulty in accessing tail vein, (2) poor blood flow leading to clot and (3) increased blood viscosity, making it difficult to mix the sample with EDTA, leading to increased hemolysis (Fig. 5C).

***In UEF and UCLA***, mean plasma absorbances in tube A or in tube B did not differ between the groups 1–6 (Tube A: UEF, p = 0.29; UCLA, p = 0.13 and tube B: UEF, p = 0.16; UCLA, p = 0.061).

***In Monash***, the absorbance differed between the groups 1–6 in tube A (p < 0.05), but not in tube B (p = 0.12). In tube A, the highest absorbance was in the earlier group 2 (0.12 ± 0.05) and the lowest in group 4 (0.07 ± 0.05). (Fig. 5C).

### 3.8. MRI follow-up

Next, we assessed the percentage of rats that completed all in vivo and ex vivo MRI timepoints as well as inter-site variability in imaging timepoints and imaging modalities.

#### 3.8.1. Percentage of rats completing in vivo MRI

The in vivo MRI was performed in the MRI cohort only. Of all 264 rats recruited into the MRI cohort, 138 (52%; 120 included in the analysis cohort, 18 excluded) were imaged at each of the four time points, 10 (4%) at 3 time points, 3 (1%) at 2 time points, 7 (3%) at 1 time point and 106 (40%) were not imaged (excluded). Of the 121 rats included in the final analysis cohort, 120 (99%) were imaged. Of the 143 rats excluded from the final study cohort, 18 (13%) were imaged (Table 3).

##### UEF.

Of the animals included in the final analysis cohort, 43/43 (100%) were imaged. In addition, 11/55 (20%) of the animals excluded from the final cohort were imaged at all timepoints.

##### Monash.

Of the animals included into the final study cohort, 41/41 (100%) were imaged at all time points (p > 0.05 as compared to UEF). In addition, 2/44 (5%) of the excluded cases were imaged at each timepoint.

##### UCLA.

Of the animals included into the final study cohort, 36/37 (97%) were imaged at all timepoints (p > 0.05 as compared to UEF or Monash). In addition, 5/44 (11%) of the rats excluded from the final analysis were imaged at all time points.

The percentage of rats imaged at all four time points and included into the final study cohort did not differ between the sites (p = 0.32).

#### 3.8.2. Percentage of rats completing ex vivo MRI

The ex vivo imaging was performed at the end of the 7-months follow-up in both the MRI and EEG cohorts.

##### MRI cohort.

Of the 117 cases included into the final MRI analysis cohort (all study sites combined), 108 (92%) were ex vivo imaged in the end of the 7-month follow-up.

Of the rats included in the final MRI analysis cohort at different study sites, 41/43 (95%) were ex vivo imaged in UEF, 41/41 (100%) in Monash and 26/33 (79%) in UCLA (p < 0.01).

Of the excluded animals, 11/44 (20%) were ex vivo imaged in UEF, 1/44 (2%) in Monash and 1/44 (2%) in UCLA.

##### EEG cohort.

Of the 124 cases included into the final EEG analysis cohort (all study sites combined), 123 (99%) were ex vivo imaged in the end of the follow-up.

Of the rats included into the final EEG analysis cohort at different study sites, 57/57 (100%) were ex vivo imaged in UEF, 43/43 (100%) in Monash and 23/24 (96%) in UCLA.

Of the excluded animals, 8/29 (28%) were ex vivo imaged in UEF, 1/65 (2%) in Monash and 2/42 (5%) in UCLA.

#### 3.8.3. Variability in timing of in vivo and ex vivo MRIs

**Fig. 6.**
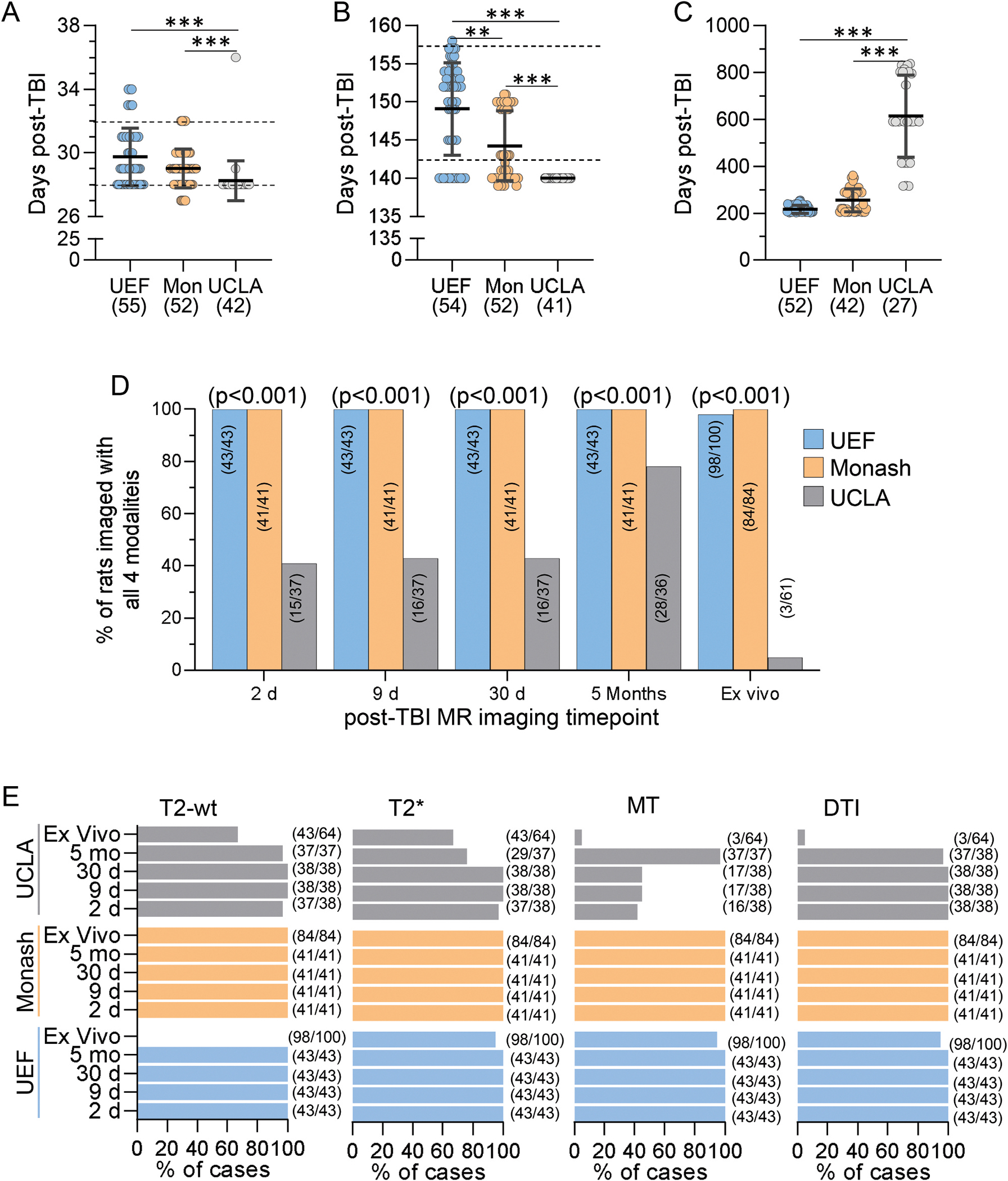
MR imaging timepoints and modalities in the final analysis cohort. Timing of (A) D30 and (B) 5 months in vivo MRIs differed between the study sites. The dashed lines indicate the pre-determined allowed deviation ( ± 2 d on D30 and ± 7 d at 5 months). (C) Also, the timing of ex vivo MRIs differed between the study sites. Note in UCLA the long duration of over 400 d after TBI (over 200 days after the 7 months follow-up period). (D) Percentage of rats in the final analysis cohort that were in vivo and/or ex vivo imaged at different time points using all four imaging modalities (T2-wt. T2 * , MT and DTI). In UCLA, on D2, D9 and D30 < 40% of the cases were in vivo imaged with all four modalities. Both cohorts were ex vivo imaged at the end of the 7 months follow-up. Note that only 3 modalities (T2 * , MT and DTI) were used for ex vivo MR imaging in UEF. (E) The percentage of rats imaged with different modalities at various timepoints. In UCLA, the MT imaging was performed to a lesser extent than at other sites. Also, only 3 rats underwent ex vivo DTI MRI. Data in (A) to (C) are presented as mean ± SD. Statistical significances: *p < 0.05, * *p < 0.01, * **p < 0.001 (Mann Whitney test).

There were no site differences in timing of the D2 and D9 in vivo MRIs. However, there was a site difference in the timing of D30 (p < 0.001) and 5 months (p < 0.001) in vivo MRIs. Also, there was a site difference in the timing of the ex vivo MRI that was scheduled to the end of the 7-months follow-up (p < 0.001) (Fig. 6A–C).

#### 3.8.4. MR imaging modalities

Next, we assessed the percentage of rats imaged with all 4 in vivo MRI pulse sequences (T2-wt, T2 * , MT and DTI) at all 4 scheduled in vivo imaging timepoints as well as with ex vivo imaging (T2-wt, T2 * , MT and DTI). We focused the analysis on the cases that were epilepsy-phenotyped and included in the final analysis cohort.

##### UEF.

Of the 43 rats included into the final UEF MRI analysis cohort, all 43 (100%) were in vivo imaged using all 4 pulse sequences at each of the 4 scheduled timepoint. Also, all 57 rats included in the final UEF EEG cohort underwent ex vivo MRI with T2 * , MT and DTI pulse sequences. The T2-wt imaging pulse sequence was excluded from ex vivo imaging (Fig. 6D).

##### Monash.

Of the 41 rats included into the final Monash MRI cohort, 41 (100%) were in vivo imaged using all 4 pulse sequences at each of the 4 scheduled timepoint. Also, all 43 rats included in the final Monash EEG cohort underwent ex vivo MRI with all 4 (T2-wt, T2 * , MT and DTI) pulse sequences (Fig. 6D).

##### UCLA.

Of the 37 rats included in the final UCLA MRI cohort, 15/37 (41%) were in vivo imaged on D2, 16/37 (43%) on D9, 16/37 (43%) on D30 and 29/37 (78%) at 5 months using all 4 imaging pulse sequences. Of the 24 animals included in the final UCLA EEG cohort, 1/24 (5%) were imaged ex vivo with all 4 pulse sequences (Fig. 6D–E).

### 3.9. Video-EEG follow-up

Next, we assessed the variability between the study sites in the number of days from TBI to electrode implantation, percentage of rats that completed all HD-EEG recording timepoints and the duration of the final phenotyping video-EEG recording (note, UCLA did not have video with the EEG).

#### 3.9.1. Percentage of rats implanted with electrodes

Pooled data from all study sites show that electrodes were implanted in 327/524 (62%, 145 in MRI and 182 in EEG cohort) of the rats initially recruited into the study. Of the implanted animals, 244/327 (121 in MRI and 123 in EEG cohort) were included into the final analysis cohort and 83/327 (24 in MRI and 59 in EEG cohort) were excluded (see Table S2 for details).

In both the MRI and EEG cohorts, the percentages of rats with electrode implantation and epilepsy-phenotyping using video-EEG monitoring were comparable (100%, note that UCLA did not perform video monitoring).

#### 3.9.2. Delay from impact to electrode implantation

##### MRI cohort.

The delay from impact to electrode implantation varied between the sites (p < 0.001), being 165 ± 10 d (range 156 – 195 d, n = 54) in UEF, 185 ± 27 d (range 142 – 239 d, n = 52) in Monash (p < 0.001 as compared to UEF) and 290 ± 57 d (range 197 – 379 d, n = 39) in UCLA (p < 0.001 as compared to UEF or Monash).

##### EEG cohort.

In UEF and UCLA, the electrodes were implanted right after the fluid-percussion injury. In Monash, electrodes were implanted 1 d after TBI in 62/75 (83%) of the rats.

#### 3.9.3. Percentage of rats with electrode re-implantation

Next, we assessed the stability of the electrode headsets by calculating the percentage of rats with electrode headset re-implantation at different study sites.

The percentage of rats that lost the headset varied between the sites (p < 0.001, χ^2^ test). In UEF, 4/131 (3%, 0 in MRI and 4 in EEG cohort) were re-implanted. In Monash, 22/126 (18%, 8 in MRI and 14 in EEG cohort) were re-implanted (p < 0.001 as compared to UEF, χ^2^ test). In UCLA, 22/66 (33%, 0 in MRI and 22 in EEG cohort) were re-implanted (p < 0.001 as compared to UEF; p < 0.05 as compared with Monash, χ^2^ test).

#### 3.9.4. Percentage of rats followed-up with high-density (HD) video-EEG monitoring

The pooled data from all three study sites show that 109/260 (42%) of the rats recruited into the EEG cohort completed all six HD-EEG recording timepoints. Of all 124 rats included into the final analysis cohort, 103 (83%) completed all six timepoints. Of the 136 excluded cases, 6 (4%) completed HD-EEG monitoring at all timepoints (Table 3).

##### UEF.

Altogether 62/86 (72%, 56 included and 6 excluded cases) of all rats recruited into the EEG cohort completed all six HD-video-EEG recording timepoints. All the rats included in the final analysis cohort, except for 1 (56/57), completed all recording timepoints.

##### Monash.

Altogether 42/108 (39%, 42 included and 0 excluded cases) of all rats recruited into the EEG cohort completed all six HD-video-EEG recording timepoints. All the rats included in the final analysis cohort, except for 1 (42/43), completed all recording timepoints (p > 0.05 as compared to UEF).

##### UCLA.

Altogether 5/66 (8%, 5 included and 0 excluded cases) of all rats recruited into the EEG cohort completed all six HD-EEG recording timepoint. Thus, 5/24 (21%) of the rats included in the final analysis cohort completed all recording timepoints (p < 0.001 as compared to UEF; p < 0.05 as compared to Monash, Table 3).

#### 3.9.5. Percentage of rats phenotyped using video-EEG recording

Data pooled from all experimental sites show that 263/521 (51%, 241 included and 22 excluded cases) of the rats recruited into the study underwent the 7th month EEG-phenotyping. Most of the post-recording exclusions (19/22, 86%) were due to a poor-quality EEG (Table 2).

##### UEF.

Altogether 99/100 (99%) of the rats recruited into the study were video-EEG-phenotyped for 30 d. One rat in the EEG cohort died of a seizure under a video-EEG monitoring on the first week of the 30-d phenotyping video-EEG on the 7th month.

##### Monash.

Altogether 84/84 (100%) of the rats recruited into the final study cohort were video-EEG-phenotyped.

##### UCLA.

Altogether 61/61 (100%) of the rats recruited into the study were EEG-phenotyped. Note that no video data was collected in UCLA.

#### 3.9.6. Duration of video-EEG recording

Our objective was to monitor for 30 d on the 7th post-injury month. The mean duration of phenotyping video-EEG recording deferred between the sites (p < 0.001). In UEF, the duration of vEEG-monitoring was 39 ± 13 d (range 9 – 69 d, n = 117). In Monash, the duration of vEEG monitoring was 38 ± 9 d (range 24 – 77 d, n = 85; p > 0.05 as compared to UEF). In UCLA, the duration of EEG monitoring was 25 ± 5 d (range 12 – 33 d, n = 64; p < 0.001 as compared to UEF or Monash).

### 3.10. Prevalence of epilepsy and characteristics of the epilepsy cohort

#### 3.10.1. Prevalence of epilepsy

The goal of the EpiBioS4Rx Project 1 was to generate a population of injured rats for biomarker discovery, of which about 25% had epilepsy.

In the final analysis cohort (MRI and EEG cohorts at all sites combined), 41/187 (22%) of the TBI rats had epilepsy (Fig. 2A–B, Table S1). The percentage of rats with epilepsy (TBI+) did not differ between the sites (UEF 21%, Monash 22%, UCLA 23%; p > 0.05, χ^2^ test).

In the MRI cohort, the rate of epilepsy was comparable at different study sites (UEF 28%, Monash 29%, UCLA 22%; p = 0.82).

Also, in the EEG cohort, the rate of epilepsy was comparable between the sites (UEF 16%, Monash 16%, UCLA 24%; p = 0.77, χ^2^ test).

#### 3.10.2. Body weight progression – TBI+ vs. TBI− rats

**Fig. 7.**
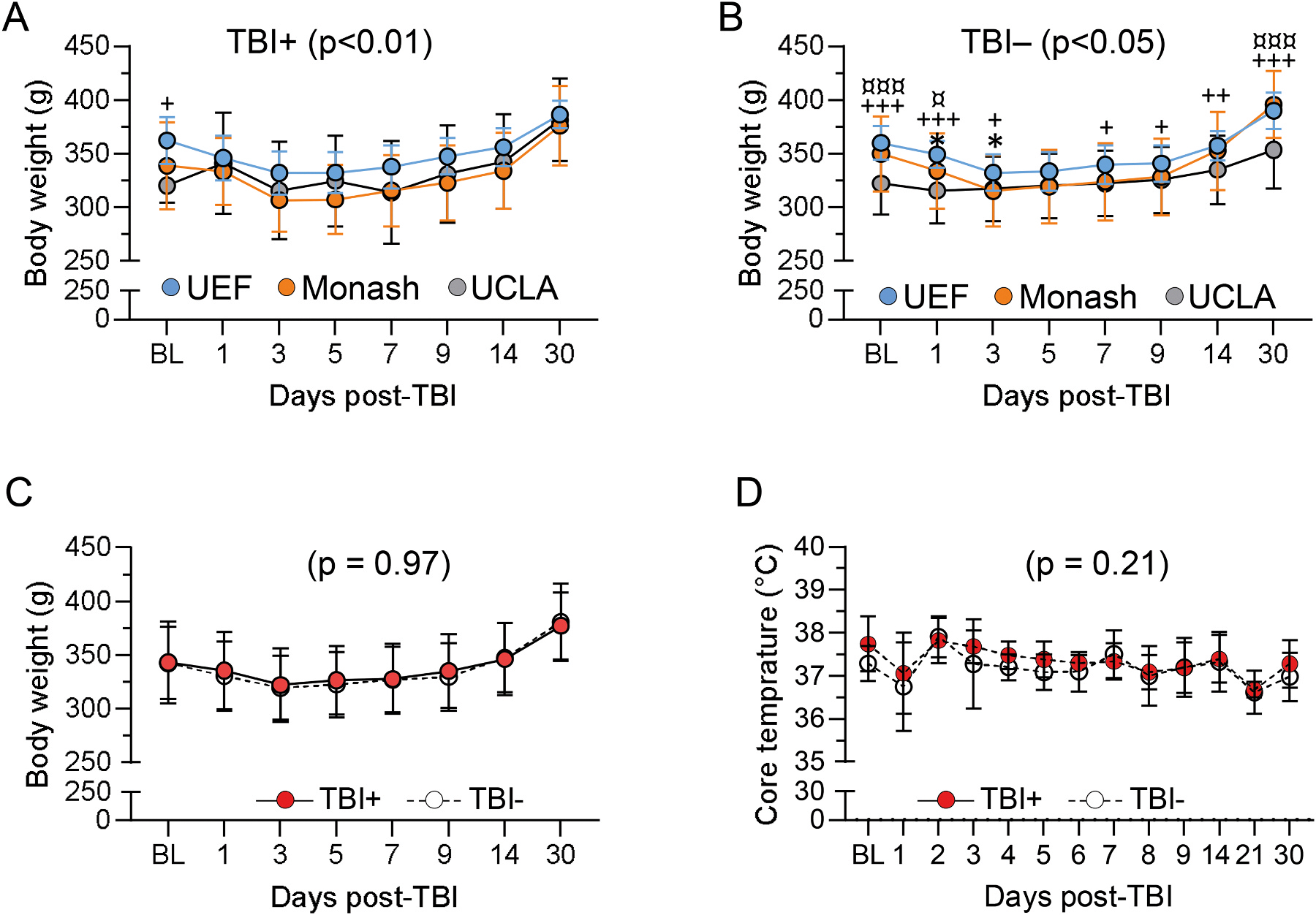
Parameters related to physiological monitoring, in rats that developed (TBI+) or did not develop (TBI−) epilepsy. (A) The post-TBI body weight progression of TBI+ rats were comparable between the sites. (B) However, there was significant difference between the sites in the progression of body weight of the TBI− rats. (C) Pooled data from all study sites revealed no differences between the TBI+ and TBI− groups. (D) Post-TBI core temperature was assessed only in UEF. There was no difference in the core temperature between sham and TBI rats or between TBI+ and TBI− rats. Data are presented as mean ± SD. Statistical significances: Panels A,B: *p < 0.05, * *p < 001, * **p < 0.001 UEF as compared to Monash; +p < 0.05, ++p < 0.01, +++p < 0.001 UEF as compared to UCLA; and ¤p < 0.05, ¤¤p < 0.01, ¤¤¤p < 0.001 Monash as compared to UCLA (Tukey’s multiple comparison test).

The progression of post-TBI body weight was comparable between TBI+ and TBI− rats at all study sites (UEF, p = 0.83; Monash, p = 0.77 and UCLA p = 0.51). The weight progression varied between the sites in the TBI+ rats (F(2, 26) = 7.9, p < 0.01) (Fig. 7A) and TBI− rats (F(2, 107) = 4.4, p < 0.05) (Fig. 7B). However, Pooled data show no difference between TBI+ and TBI− rats (p = 0.97) (Fig. 7C).

#### 3.10.3. Core body temperature – TBI+ vs. TBI− rats

In UEF, the core temperature was comparable between the TBI+ and TBI− rats at all timepoints (p = 0.21) (Fig. 7D). Note that core temperature was not assessed at other sites.

#### 3.10.4. Impact pressure – TBI+ vs. TBI− rats

##### Whole TBI cohort.

**Fig. 8.**
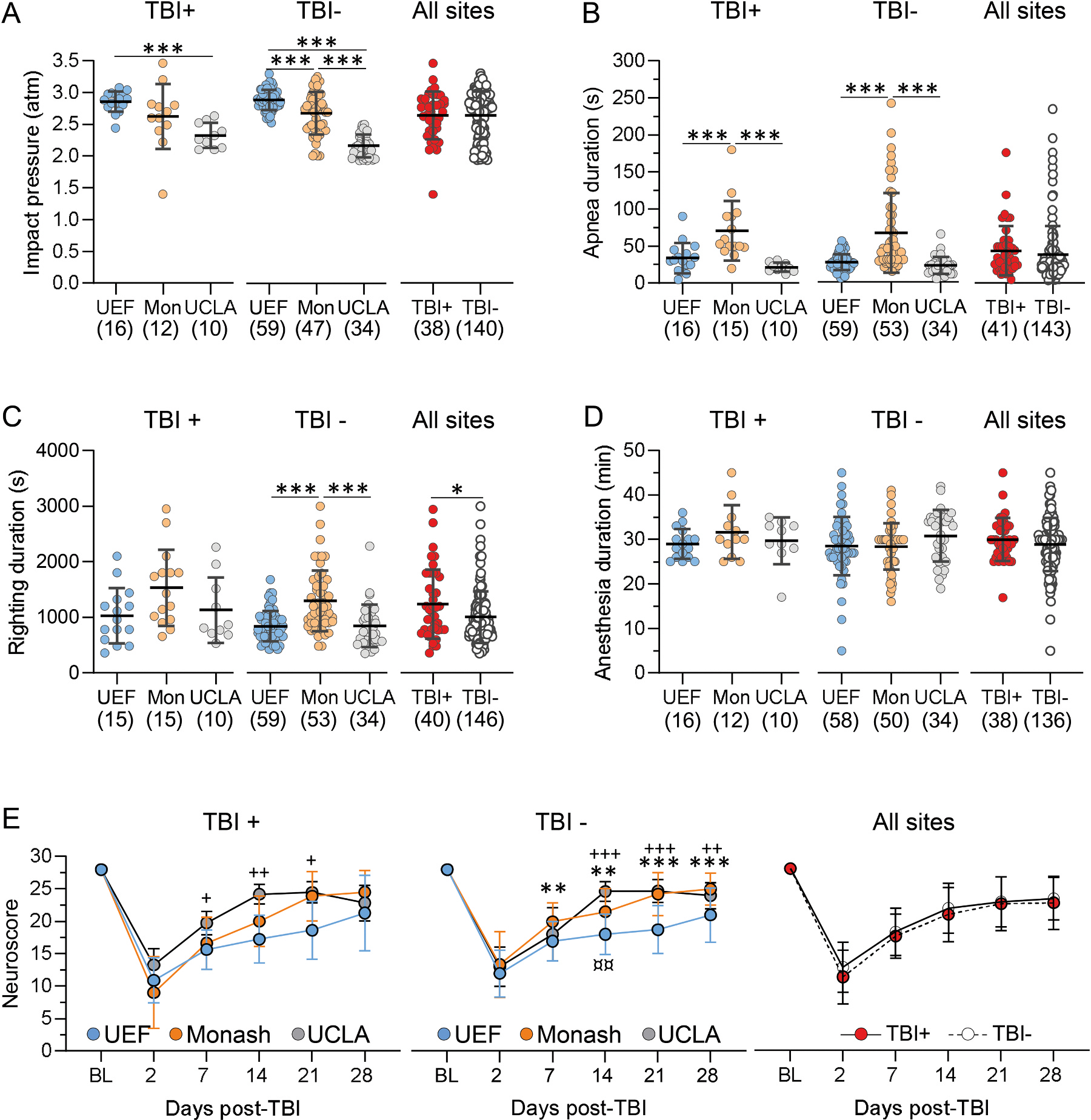
Parameters related to TBI induction and acute somato-motor deficits in rats that developed (TBI+) or did not develop (TBI−) epilepsy. (A) The mean impact pressure in the TBI+ or TBI− groups differed between sites (p < 0.001). There was no difference between the TBI+ and TBI− groups when data from all sites was pooled together (p = 0.48). (B) Also, the mean post-impact apnea duration in the TBI+ or TBI− groups differed between sites (p < 0.001). There was no difference between the TBI+ and TBI− groups when data from all sites was pooled together (p = 0.10). (C) The time to self-right in the TBI+ group did not differ between the sites but showed site-differences in the TBI− groups (p < 0.001). When data from all sites was pooled, the time to self-right was longer in the TBI+ than TBI− group (p < 0.05). (D) The average duration of anesthesia during the TBI surgery was comparable at different sites both in the TBI+ and TBI− group. When data from all sites was pooled, anesthesia duration was comparable between the TBI+ and TBI− groups (p > 0.99). (E) A mixed-effects model analysis showed that the evolution of the neuroscore in the TBI+ (p < 0.01) and TBI− (p < 0.001) varied between the sites. However, when data from all sites was pooled, the performance of the TBI+ and TBI− rats was comparable. Data are presented as mean ± SD. Statistical significances: Panels A-D: *p < 0.05, * *p < 0.01, * **p < 0.001 (Mann-Whitney U test); Panel I: *p < 0.05, * *p < 001, * **p < 0.001 UEF as compared to Monash; +p < 0.05, ++p < 0.01, +++p < 0.001 UEF as compared to UCLA; and ¤p < 0.05, ¤ ¤p < 0.01, ¤¤¤p < 0.001 Monash as compared to UCLA (Tukey’s multiple comparison test); Abbreviations: Mon, Monash.

Impact pressure did not differ between the TBI+ and TBI− rats (p = 0.48) (Fig. 8A).

##### TBI+.

The mean impact pressure in the TBI+ cohort (TBI+ rats in the MRI and EEG cohorts combined) varied between the sites (p < 0.001). The impact pressure in UEF was higher than that in UCLA (p < 0.001) (Fig. 8A).

##### TBI−.

The mean impact pressure in the TBI− cohort also varied between the sites (p < 0.001). The impact pressure in UEF was higher than that in Monash (p < 0.01) or UCLA (p < 0.001). The impact pressure in Monash was higher than that in UCLA (p < 0.001, Fig. 8A).

##### *TBI*+ vs. *TBI*−.

In UCLA, the impact pressure in the TBI+ group (2.3 ± 0.19 atm, range: 2.1 – 2.6 atm, n = 10) was higher than in the TBI− group (2.2 ± 0.18 atm, range: 1.9 – 2.5 atm, n = 34) (p < 0.05). The pressure was comparable between the groups in UEF (p = 0.87) and Monash (p = 0.71).

#### 3.10.5. Post-impact apnea – TBI+ vs. TBI− rats

##### Whole TBI cohort.

The duration of post-impact apnea did not differ between the TBI+ and TBI− rats (p = 0.10) (Fig. 8B).

##### TBI+.

The mean apnea duration in the TBI+ cohort (TBI+ rats in the MRI and EEG cohorts combined) varied between the sites (p < 0.001). The duration was longer Monash as compared to that in UEF (p < 0.01) or UCLA (p < 0.001) (Fig. 8B). There was no difference in the duration between UEF and UCLA.

##### TBI−.

The mean apnea duration also varied between the sites (p < 0.001). The longest duration was observed in Monash as compared to UEF (p < 0.001) and UCLA (p < 0.001) but was comparable in UEF and UCLA (p < 0.01) (Fig. 8B).

##### *TBI*+ vs. *TBI.*

Pooled data (MRI and EEG cohort combined), show that duration of post-impact apnea was comparable in the TBI+ and TBI− groups in all the sites (UEF, p = 0.14; Monash, p = 0.13 and UCLA, p = 0.73).

#### 3.10.6. Righting reflex – TBI+ vs. TBI− rats

##### Whole TBI cohort.

The mean time to self-right after TBI was higher in the TBI+ than TBI− groups (p < 0.05) (Fig. 8C). A receiver operator characteristic (ROC) analysis showed that the groups could only be marginally differentiated with an area under the curve (AUC) of 0.606 (p < 0.05). A cut-off value of 1 623 s will identify only 28% of the true positives and 8% of the true negatives.

##### TBI+.

The mean duration of time to self-right did not differ between the sites (p = 0.093) (Fig. 8C).

##### TBI−.

The righting reflex time varied between the sites (p < 0.001). The righting time in Monash was longer than that in UEF (p < 0.001) or UCLA (p < 0.001). The righting times were comparable in UEF and UCLA (p = 0.73) (Fig. 8C).

##### *TBI*+ vs. *TBI−*.

Time to self-righting after impact was comparable between TBI+ and TBI− groups in all the sites (UEF, p = 0.57; Monash, p = 0.99. and UCLA, p = 0.56).

#### 3.10.7. Duration of anesthesia during TBI surgery – TBI+ vs. TBI− rats

##### Whole TBI cohort.

The duration of exposure to anesthesia during TBI surgery did not differ between the TBI+ and TBI− rats (p > 0.99) (Fig. 8D).

***TBI***+ ***or TBI****−* ***groups,*** the duration of anesthesia did not differ between the sites (TBI+, p = 0.35; TBI−, p = 0.088) (Fig. 8D).

##### *TBI*+ vs. *TBI−*.

Duration of exposure to anesthesia was comparable in the TBI+ and TBI− groups in all the sites (UEF, p > 0.99; Monash, p = 0.66; UCLA, p > 0.99).

#### 3.10.8. Composite neuroscore – TBI+ vs. TBI− rats

##### Whole TBI cohort.

The combined data from all sites showed that the neuroscore was comparable between TBI+ and TBI− (p = 0.087). However, there was a difference in neuroscore between the time points in each group (F(3.9, 411) = 285, p < 0.001) (Fig. 8E).

##### TBI+.

There neuroscore differed between the sites (F_(2, 26)_ = 7.88), p < 0.01). Moreover, there was a difference in the time point (F_(4, 100)_ = 123, p < 0.001) with an interaction between site and time point (F_(10, 127)_ = 3.8, p < 0.001) (Fig. 8E).

##### TBI−.

There was also a difference in the neuroscore between the sites (F_(2, 123)_ = 13.8, p < 0.001) and a difference between the time points (F_(4, 425)_ = 269, p < 0.001) with an interaction between the site and time point (F_(10, 603)_ = 4.19, p < 0.001) (Fig. 8E).

##### *TBI*+ vs. *TBI−*.

In UEF, there was no difference in the neuroscore between the groups (F_(1, 30)_ = 0.67, p = 0.42). However, the neuroscore differed between the time points in each group (F_(4, 112)_ = 102, p < 0.001) with an interaction between the time point and site (F_(5, 150)_ = 0.29, p < 0.001). In Monash, the neuroscore did not differ between the groups (F_(1, 29)_ = 3.89, p = 0.06) but differed between the time points (F _(3, 68)_ = 85, p < 0.001) with no interaction between the group and the time point (F_(5, 136)_ = 1.44, p = 0.21). In UCLA, there was no difference between the groups ( F_(1, 45)_ = 0.007, p = 0.93), but it also varied between the time points (F_(3, 131)_ = 179, p < 0.001) with no interaction between the group and time point (F_(5, 225)_ = 1.5, p = 0.19).

## 4. Discussion

The aim of the Epibios4Rx Project 1 was to identify prognostic plasma, EEG and MRI biomarkers for epileptogenesis after TBI in the rat FPI model. To ensure statistical power, an international multicenter study was designed. This required the participating centers in Finland (UEF), Australia (Monash) and USA (UCLA) to make rigorous attempts at harmonizing the methodological protocols, data collection and data analysis using CDEs over the 3-y study period as well as adjustment of pre-defined protocols when needed. In this study we assessed the success of our procedural harmonization. We had four major findings: (1) all experimental sites were able to adhere to the predetermined procedures even though there was some variability in the procedural coverage applied, (2) the blood sampling and MRI timepoints showed the most variability, (3) decrease in acute mortality and increase in plasma quality across time reflected a learning effect in the TBI production and blood sampling protocols, (4) the prevalence of epilepsy at different study sites was comparable, and was not influenced by some inter-center procedural variability.

### All sites produced rats with moderate to severe TBI despite of some procedural variability.

Severity of TBI has been shown to be the most critical single factor associated with the development of PTE both in animal models and humans (Annegers et al., 1998; Annegers and Coan, 2000; Kharatishvili et al., 2006). Therefore, a key aspect of our harmonization of protocol was the production of rats with severe TBI to maximize the prevalence of epilepsy after a 7-month follow-up.

The main parameters used to assess the severity of TBI were the acute mortality and the impact pressure (Kharatishvili et al., 2006; McIntosh et al., 1989). Acute mortality was anticipated to be around 30%. However, it varied significantly between the sites, being up to 40% in UCLA with the lowest average impact pressure and as low as 4% in UEF with the highest impact pressure. This suggests in the EpiBioS4Rx studies, acute mortality is not a strong indicator of injury severity and could indicate other factors affecting injury severity (e.g., methodological differences in injury production, gene-related susceptibility differences between Sprague Dawley rats from different vendors (Kristensen et al., 2017). Moreover, it was assumed that in consortia like the EpiBioS4Rx, the procedures could be relatively easily reproduced by other sites using the same experimental protocols, materials and inter-center training. However, some of the intra-site and inter-site variability in the TBI production parameters could have related to the fact that UCLA had just started to perform the lateral FPI model. Also, unlike in UEF where the same technician performed all injuries, in UCLA and Monash more than one person was involved in TBI surgeries and electrode implantation. Our experience suggest that procedural harmonization may be more challenging than anticipated, especially when using an animal model that requires complex surgical protocols, which was proposed to require over 100 h of practice to attain expertise (Garijo et al., 2013; Llovera et al., 2015).

To minimize the device-related variability in impact generation, all study sites purchased their fluid-percussion device from the same vendor. The planned impact pressure for induction of PTE was aimed at around 2.8 atm. As our data show, the slight variability in impact pressure did not affect epileptogenesis, which was comparable between the three study sites. Moreover, impact pressures within the TBI+ cohort varied from 1.5 to 3.5 atm that could represent differences between pressure recorded at the tip of device and actual pressure impacting the brain.

The other independent parameters used to monitor severity of TBI included duration of post-impact apnea, righting reflex time and composite neuroscore. Importantly, variability in these parameters between the sites did not affect the prevalence of epileptogenesis. The D2 neuroscore varied between 7 and 17, indicating the induction of severe to moderate injury (Pitkänen and McIntosh, 2006). In correlation with our preliminary findings, the average impairment in D2 neuroscore did not differ between the study sites. However, we found site differences in recovery of acute somato-motor deficits (Ndode-Ekane et al., 2019). Somewhat unexpectedly, we found inter-site variability in neuroscore of sham-operated rats. This may reflect the subjective nature and the extent of prior experience in the assessment of neuroscore.

In summary, all sites produced rats with moderate to severe TBI despite marginal difference in the impact pressure, site-specific difficulty in the production of the TBI model and subjective differences in the assessment of post-TBI acute somato-motor deficits.

### 4.1. Experimental sites adhered strictly to systematic data collection and procedural timing accuracy but the study population coverage varied

We assessed three major axes of procedural harmonization: (a) performance (yes/no) of the procedures, which is critical for data availability, (b) accuracy in timing of the procedure, which is critical for time-specific biomarker analysis and (c) percentage of the animals undergoing the planned procedures, which is critical to maintain the statistical power of the biomarker analysis.

#### Weight.

The frequent assessment of body weight served as an indirect indicator of post-TBI recovery and adherence to animal license restrictions related to animal welfare and exclusions. Also, post-injury body weight was recently suggested as a potential biomarker for epileptogenesis, and thus, considering its role in the final biomarker analysis (Lapinlampi et al., 2020). Initially a daily weight monitoring was planned over the first 2 weeks, and weekly or monthly thereafter. However, in UEF and Monash less than 60% of all rats recruited into the study were weighted at each timepoint, including the rats in the MRI cohort only. To reduce the risk of loss of electrode headset, animals in the EEG cohort were weighted only at baseline or when the rat was suspected of having weight loss. In UCLA, all included cases in both cohorts were weighted, but only in every other of the pre-scheduled time points to reduce the workload with limited manpower. Interestingly, the percentage of animals with the loss of headsets in the EEG cohort over the follow-up was higher in UCLA as compared to that in UEF or Monash, the daily animal handling being one of suspected headset destabilizing factor. Overall, 31% of the rats in the final analysis cohort had a complete weight monitoring. The lesson learned was that the practices need to be regularly monitored to justify the need of protocol changes during the course of chronic EEG studies, requiring a stable and well-positioned electrode implantation.

#### Plasma.

Sampling of tail vein blood and plasma preparation procedures were practiced by the consortium members (Kamnaksh et al., 2019). By using the defined procedures, 64% of all the rats recruited to the study were sampled on D2 and D9. The deviation in timing of the sampling on D2 was from − 7 to + 7 h and on D9 from − 7 to + 7 h, indicating that a large number of representative samples would be available for plasma biomarker analysis at these early time points (Dadas et al., 2018; Munoz Pareja et al., 2022; Petersen et al., 2021). Timing of D30 and 5 months samples showed some variability. This was expected as the study protocol allowed a ± 2 d and ± 7 d deviations, respectively. Other reasons contributing to variability in timing are related to logistics of handling a larger animal number within a short time window with limited human resources and availability of materials like cold centrifuges and shared workspace. Overall, 94% of the rats in the final analysis cohort had a complete set of plasma samples for analysis.

#### MRI.

In preclinical setting, harmonization procedures for MRI imaging remains a challenge. Our preliminary analysis demonstrated that all study sites were able to perform all planned imaging pulse sequences (Immonen et al., 2019). All centers adhered to use the planned imaging modalities. Overall, 85% of the rats included in the final analysis cohort were imaged using all four pulse sequences in in vivo MRI. In UCLA, however, the MT pulse sequence was performed only in 58% of the animals. This related to early hardware inconsistencies that existed initially at UCLA that were specific to the MT sequence parameters. The complete set of ex vivo MRIs with 4 pulse sequences was performed in 36% of rats in the final analysis cohort. In UCLA, the MT and DTI pulse sequences was used only in 5% of animals during the ex vivo imaging. In UEF, T2-wt was not done as planned in the original ex vivo MRI protocol, as multi echo gradient echo (average over echoes) provided similar anatomical information. Nonetheless, UCLA and Monash did add T2. The deviation in timing of MRI on D2 and D9 was small. More variability was found in the timing of D30 and 5 months MRIs which was expected as the study protocol allowed ± 2 d and ± 7 d deviations for these time points, respectively. Overall, 82% of the rats included in the final analysis cohort had a complete set of in vivo and ex vivo MRI datasets.

#### EEG.

The major gaps in harmonization related to the post-TBI monthly HD video-EEG recordings. In UCLA, no video was available, and consequently, posttraumatic seizures could only be characterized electrographically without behavioral correlates. The decision to omit the video in UCLA was reached after it became clear that simultaneous monitoring of 20 + animals needed for a timely performance of the experiments was not feasible in UCLA as it had required modification of the monitoring room infrastructure and the Intan-based EEG system used to acquire the data. However, all diagnostic seizures were reviewed by investigators from all three centers and agreed. Therefore, the lack of video monitoring did not affect the diagnosis of PTE. Second, in UCLA less than 50% of the included cases were monitored with the monthly HD-EEG after the second month recording session. This related to the loss of electrode headsets, and consequently, elimination of further depth electrode recordings as it was predetermined to reimplant the headset only once and with the epidural electrodes only. Whether the lower than anticipated number of rats with HD-EEG follow-up will affect the robustness of any potential EEG biomarker remains to be determined. In Monash, the animals in the EEG cohort had depth and epidural electrode implantations after TBI. However, majority of the rats in the MRI cohort had epidural electrodes only, which limited the delineation of the seizure onset site, but not the epilepsy diagnosis. Need of training and monitoring of electrode implantation procedures will be necessary in studies requiring chronic EEG recordings as noted also in other studies (Medlej et al., 2019).

Taken together, our data shows adherence by all experimental sites to the follow-up procedures and procedural timepoints set out in study design and marking the protocol deviations. Moreover, despite sites differences in study population and technical difficulties, the data suggest a clear harmonization of the follow-up procedures between the participating sites.

### 4.2. The prevalence of epilepsy was not influenced by variability in TBI production parameters or duration of 7th month EEG recording

The present study was powered based on previous studies showing approximately a 25% epilepsy prevalence 6–7 months after lateral FPI (Kharatishvili et al., 2006; Shultz et al., 2013). In the present study the average prevalence of PTE in the final analysis cohort was 22% when both the MRI and EEG cohorts were combined. Thus, we were able to achieve the statistical power as anticipated in original power calculations.

However, there are some issues that may bias the presentation of different sites in the final analysis cohort. The number of TBI+ rats produced by each site varied, being the lowest by UCLA. This related to a low number of rats initially recruited to the study, including exclusion of rats (n = 10) that were in progress at the time of recruitment end date, and higher than anticipated acute mortality. Consequently, 39% of the rats in the TBI+ cohort were produced by UEF, 37% by Monash and 24% by UCLA. The respective percentage in non-epileptic TBI− animals were 41% in UEF, 36% in Monash and 23% in UCLA. Another bias may relate to a shorter duration of the 7th month EEG recordings for epilepsy phenotyping. In UCLA the duration of EEG monitoring was on average 13 d shorter than in UEF and Monash, which could misclassify some TBI− animals.

### 4.3. Heterogeneity of rats with PTE

Next, we analyzed the difference between the TBI+ and TBI− animals in acutely monitored parameters. We assumed them to be comparable, and thus, not to influence D2 and D9 plasma analysis or MRI imaging. As shown earlier, impact pressure and apnea duration did not differ between the TBI+ and TBI− rats, suggesting that the rate of epilepsy was independent of these variables. Interestingly, the righting reflex time was longer in the TBI+ than TBI group when the data from all sites was combined. However, ROC analysis indicated that the strength of the righting reflect time to differentiate between the TBI+ and TBI− groups was poor. Moreover, there was no difference in recovery of acute somato-motor deficits between the TBI+ and TBI− groups at any study site. However, comparison of TBI+ or TBI− rats between the sites showed differences in the recovery. Even though this may relate to subjective differences in the assessment of neuroscore, the variable speed of recovery of pathologies underlying the post-injury functional improvement may have an influence on expression of molecular and tissue biomarkers.

The post-TBI body weight progression in the TBI+ and TBI− groups was assessed as a biomarker for epileptogenesis as previously suggested (Lapinlampi et al., 2020). However, the body weight of the animals included in the final analysis cohort differed between TBI and sham-operated rats only in UEF. This was unexpected as post-injury weight loss is a widely reported phenomenon in lateral FPI model (Allioux et al., 2022; Lapinlampi et al., 2020). One explanation could relate to differences in the post-operative food supplements. In Monash, rats were fed with powder milk. In UCLA, rats received food pellets infused with anti-inflammatory agent (Flu-Nix) and antibiotics. Administration of nonsteroidal anti-inflammatory agents after experimental TBI can indirectly influence the body weight as animals recover better and eat more frequently (Anderson et al., 2021). These protocol differences in Monash were recommended and in UCLA, Flu-Nix was required, by the local animal welfare ethics committee. Consequently, when data from all sites were combined, the body weight progression was comparable between the TBI+ and TBI− groups.

In summary, the rate of epilepsy was the same between experimental sites despite differences in TBI production parameters as well as in recovery of acute somato-motor deficits or body weight.

### 4.4. The final study population is statistically powered based on the rate of epilepsy

Low statistical power has been considered as one of the major reasons for a poor reproducibility and translatability of preclinical research findings to clinic (Llovera and Liesz, 2016). As a cure for this major preclinical challenge, it has been suggested to design standardized and harmonized multi-center preclinical trials with sufficient statistical power. The EpiBios4Rx project was designed to produce enough animals to discover biomarkers with ROC AUC 0.700. The study was initially slightly over-powered to anticipate unforeseen loss of animals or study delays.

We achieved a 22% overall epilepsy prevalence which was 3% units less than the anticipated 25%. Interestingly, the prevalence of 25% used in power calculations was based on animal cohorts that had been produced in study designs comparable to that of the EpiBioS4Rx MRI animal cohort, in which the epilepsy rate was 27% (Lapinlampi et al., 2020). For a reason currently unknown, the prevalence of epilepsy in the EEG cohort, in which the animals had the epidural and intracerebral electrodes implanted right after the injury surgery tended to be lower as compared to that in the MRI cohort that was implanted 6 months after injury (18% vs. 27%).

In the EEG cohort, we also found a somewhat higher acute mortality and exclusion rate than expected. Moreover, we had to exclude a large number of EEG-phenotyped animals (17% of excluded population) after realizing that the EEG recorded was of very poor quality due to manufacturer-related technical problem with the 12-electrodes pogo-pin connectors that we initially used in the electrode headset. This affected all study sites as it was agreed that all EEG recording accessories, including recording electrodes, had to be purchased from the same vendor. Nonetheless, technical difficulties, especially when dealing with animal models are not uncommon in pRCTs (Llovera and Liesz, 2016).

To summarize, the project produced a study population with an effect size that is statistical powered despite site-specific difficulties in the production of the TBI model and the exclusion of large number of animals. It will be interesting to see whether future pRCT with similar objective can result in comparable epilepsy outcome.

### 4.5. Implications for discovery of biomarkers

It is evident from our data that the procedural harmonization has been successfully implemented. In spite of differences in the model production, the epilepsy outcome was the same. Ongoing analyses will determine whether some of the observed variability in procedural harmonization will impact blood, MRI and EEG biomarkers and whether the effect will be modality specific or common to all biomarker types. These findings will, however, provide insight into any observed variability in the biomarkers and allow a better structuring of analysis strategies by anticipating the effect of protocol deviations on biomarker discovery and use.

## 5. Conclusion

This study shows the feasibility, as well as challenges, of conducting multicenter preclinical studies of post-traumatic epileptogenesis. Over 80% of all rats included in the final analysis cohort were assessed at all pre-scheduled timepoints, including the assessment of composite neuroscore, tail vein blood sampling, in vivo and ex vivo MRI, and the final 30 d HD video-EEG recording, indicating a strict adherence to the follow-up procedures. Our data show that use of predefined CDEs helped the participating study sites to follow the common study design and systematically collect multimodal data for biomarker analysis. The procedural and data collection variability between the study sites was in large part due to inherent difference between the experimenters and to a lesser extent due to site-specific protocol deviations. This was not unexpected, especially when dealing with animal models and procedures which require several months of training to master them with minimal complications. Importantly, the procedural differences did not affect the prevalence of epilepsy in the final analysis cohort, indicating the reproducibility of the lateral FPI-induced PTE model. Moreover, we could demonstrate a learning effect over time that reduced the inter-site variability. The major lessons learned relate to the importance of hands-on training of all procedures, to allocation of resources on continuous follow-up of practices at different centers and *interim* analysis of protocols and data to adjust them if needed.

## Supplementary Material

Supplementary material

Appendix A. Supporting information

Supplementary data associated with this article can be found in the online version at doi:10.1016/j.eplepsyres.2023.107263.

## Figures and Tables

**Table 1:** Materials used in different procedures at different study sites.

	Site	Catalogue number	Vendor	Country

Rats	UEF	Sprague Dawley (SD)	Envigo Laboratories B.V.	The Netherlands
	Monash	Sprague Dawley (SD)	In house bred, Animal Research Platform (MARP) Monash University	Australia
	UCLA	Sprague Dawley (SD)	Charles River	USA
Induction of TBI				
*Anesthesia system*	UEF/Monash	Somnosuite # SS6069B	Kent Scientific	USA
	UCLA	Matrix VIP 3000 Vaporizer # 91305430	Patterson Veterinary	
*Trephine*	UEF/ UCLA	#18004–50	Fine Science Tools GmbH	Germany
	Monash	Dremmel Model 300	Model 300	Australia
*Tissue adhesive*	UEF/ UCLA	3 M Vetbond	3 M Deutschland GmbH	Germany
	Monash	Octyl cyanoacrylate	Bostik	Australia
*Dental acrylate*	UEF	Selectaplus #10009210, Selectaplus #D10009102	DeguDent	Germany
	Monash	AVSCV00500	Vertex	The Netherlands
	UCLA	SNAP Liquid (P16–02–65), SNAP Powder (P16–02–60)	Pearson Dental	USA
Fluid percussion device	All sites	Model FP 302	AmScien Instruments	USA
Postoperative care				
*Analgesic*	UEF	Buprenorphine	Orion Pharma	Finland
	Monash	Buprenorphine	Indivior Pty Ltd	Australia
	UCLA	Flunixin meglumine	MERK	USA
*Additional analgesic*	UEF/Monash	Not applicable		
	UCLA	Flu-Nix Flunixin Meglumine	AgriLabs	USA
*Medical oxygen*	UEF/ UCLA	Not applicable	Not applicable	Not applicable
	Monash	Not applicable	Mediquip Medical Equipment & Supplies	Australia
*Antibiotics*	UEF/Monash	Not applicable	Not applicable	Not applicable
	UCLA	Trimethoprim sulfamethoxazole (TMS) medicated rodent chow	Envigo Laboratories	USA
*Food pellet*	UEF	2016S (Teklan Diet)	Envigo Laboratories B.V.	The Netherlands
	Monash	102108	Barastoc	Australia
	UCLA	LabDiet 5001 *	LabDiet	MO, USA
*Food supplement*	UEF/ UCLA	Not applicable	Not applicable	Not applicable
	Monash	(Powder milk)	Sustagen, Nestle	Australia
Blood sampling				
*23 G butterfly needle*	All sites	#367284, BD Vacutainer	BD Biosciences	NJ, USA
*K2EDTA tube*	All sites	#365975, BD Microtainer	BD Biosciences	NJ, USA
*Centrifuge*	UEF/ UCLA	Centrifuge 5417 R	Eppendorf Biotools	CA, USA
	Monash	Centrifuge 5415 R	Eppendorf Biotools	CA, USA
*UV-vis absorbance microcentrifuge tubes*	All sites	ND-1000, NanoDrop^™^	Thermo Fisher Scientific	Wilmington, DE
	All sites	#022431064, Eppendorf^®^	Eppendorf Biotools	CA, USA
MR Imaging				
*Magnet/ console*	UEF	7 T/16 cm/ Bruker Pharmascan	Bruker Corporation	MA, USA
	Monash	4.7 T/33 cm/ Bruker Biospec	Bruker Corporation	MA, USA
	UCLA	7 T/30 cm/ Bruker Biospec	Bruker Corporation	MA, USA
Video-EGG				
*12-channel pedestal*	All sites	MS12 P EM12/20/2.4/SP	PlasticsOne Inc.	VA, USA
*Cable*	All sites	M12C-363/2	PlasticsOne Inc.	VA, USA
*Commutator*	All sites	SL12C, 12-pin swivel	PlasticsOne Inc.	VA, USA
*Amplifier model*	UEF	Digital Lynx 16SX	Neuralynx	USA
	Monash	Neuvo	Compumedics^®^	Australia
	UCLA	Intan RHD2000	Intan Technologies	CA, USA

**Table 2:** The percentage of rats excluded under different criteria. The total number of animal excluded in each site in brackets.

	UEF	Monash	UCLA	Total

Abscess	1% (1/84)	0% (0/108)	4% (3/86)	1% (4/278)
Acute mortality	29% (24/84)	46% (50/108)	71% (61/86)	48% (135/278)
Bad EEG	20% (17/84)	24% (26/108)	1% (1/86)	7% (19/278)
Broken dura	23% (19/84)	6% (6/108)	12% (10/86)	13% (35/278)
Contra Lesion	1% (1/84)	0% (0/108)	0,0% (0/86)	0.4% (1/278)
Corneal ulcer	0% (0/84)	2% (2/108)	0,0% (0/86)	1% (2/278)
Dead - reimplantation	0% (0/84)	0% (0/108)	8% (7/86)	3% (7/278)
Dead post-SE	0% (0/84)	0% (0/108)	1% (1/86)	0,4% (1/278)
Dead post-seizure	0% (0/84)	0% (0/108)	1% (1/86)	0,4% (1/278)
Device malfunction	2% (2/84)	2% (2/108)	0% (0/86)	1% (4/278)
Extra rats	18% (15/84)	0% (0/108)	0% (0/86)	5% (15/278)
Follow-up mortality	1% (1/84)	2% (2/108)	2% (2/86)	2% (5/278)
Frontal lesion	2% (2/84)	0% (0/108)	0% (0/86)	1% (2/278)
Leur-lock came off	0% (0/84)	2% (2/108)	0% (0/86)	1% (2/278)
Lost implant	0% (0/84)	6% (7/108)	0% (0/86)	3% (7/278)
Malocclusion	0% (0/84)	3% (3/108)	0% (0/86)	1% (3/278)
Tumor	0% (0/84)	1% (1/108)	0% (0/86)	0,4% (1/278)
Weight loss	3% (2/84)	7% (8/108)	0% (0/86)	4% (10/278)

**Table 3:** The percentage of rats that completed all timepoints and follow-up procedures at different study sites. Data from the MRI and EEG cohorts are shown separately. Data are shown to all rats recruited into the study as well as to animals that were included in the final analysis cohort or excluded. Note that no follow-up high-density EEG recordings were applicable in the MRI cohort (NA).

		Neuroscore	Blood sampling	MRI	Core temperature	Body weight	Monthly HD EEG

**UEF**	**MRI cohort**						
	Included	98% (42/43)	100% (43/43)	100% (43/43)	7% (3/43)	7% (3/43)	NA
	Excluded	47% (26/55)	44% (24/55)	20% (11/55)	4% (2/55)	4% (2/55)	NA
	Total	69% (68/98)	68% (67/98)	55% (54/98)	5% (5/98)	5% (5/98)	NA
	**EEG cohort**						
	Included	0% (0/57)	98% (56/57)	NA	0% (0/57)	0% (0/57)	98% (56/57)
	Excluded	0% (0/29)	24% (7/29)	NA	0% (0/29)	0% (0/29)	21% (6/29)
	Total	0% (0/86)	73% (63/86)	NA	0% (0/86)	0% (0/86)	72% (62/86)
**Monash**	**MRI cohort**						
	Included	81% (33/41)	100% (41/41)	100% (41/41)	0% (0/41)	17% (7/41)	NA
	Excluded	14% (6/44)	25% (11/44)	5% (2/44)	0% (0/44)	0% (0/44)	NA
	Total	46% (39/85)	61% (52/85)	51% (43/85)	0% (0/85)	8% (7/85)	NA
	**EEG cohort**						
	Included	2% (1/43)	100% (43/43)	NA	0% (0/43)	14% (6/43)	98% (42/43)
	Excluded	9% (6/65)	12% (8/65)	NA	0% (0/65)	0% (0/65)	0% (0/65)
	Total	7% (7/108)	47% (51/108)	NA	0% (0/108)	6% (6/108)	39% (42/108)
**UCLA**	**MRI cohort**						
	Included	100% (37/37)	81% (30/37)	97% (36/37)	0% (0/37)	100% (37/37)	NA
	Excluded	14% (6/44)	7% (3/44)	11% (5/44)	0% (0/44)	11% (5/44)	NA
	Total	53% (43/81)	41% (33/81)	51% (41/81)	0% (0/81)	52% (42/81)	NA
	**EEG cohort**						
	Included	100% (24/24)	75% (18/24)	NA	0% (0/24)	100% (24/24)	21% (5/24)
	Excluded	19% (8/42)	2% (1/42)	NA	0% (0/42)	17% (7/42)	0% (0/42)
	Total	49% (32/66)	29% (19/66)	NA	0% (0/66)	47% (31/66)	8% (5/66)
**Total**	**MRI cohort**						
	Included	93% (112/121)	94% (114/121)	99% (120/121)	3% (3/121)	39% (47/121)	NA
	Excluded	27% (38/143)	27% (38/143)	13% (18/143)	1% (2/143)	5% (7/143)	NA
	Total	57% (150/264)	58% (152/264)	52% (138/264)	2% (5/264)	21% (54/264)	NA
	**EEG cohort**						
	Included	20% (25/124)	94% (117/124)	NA	0% (0/124)	24% (30/124)	83% (103/124)
	Excluded	10% (14/136)	12% (16/136)	NA	0% (0/136)	5% (7/136)	4% (6/136)
	Total	15% (39/260)	51% (133/260)	NA	0% (0/260)	14% (37/260)	42% (109/260)

Abbreviations: EEG, electroencephalography; MRI, magnetic resonance imaging.
