# Supplementary material for: Successful harmonization in EpiBioS4Rx biomarker study on post-traumatic epilepsy paves the way towards powered preclinical multicenter studies
Source: Epilepsy Res. Author manuscript; Available in PMC 2025 Jul 28. (PMC12301999; doi:10.1016/j.eplepsyres.2023.107263)
Supplement: Supplementary material [file NIHMS2095868-supplement-Supplementary_material.docx]

**Supplementary Table 1.** Summary of cases phenotyped with a 1-month video-EEG on the 7^th^ post-injury month.

|  | **UEF** | **Monash** | **UCLA** | **Total** |
| --- | --- | --- | --- | --- |
| **MRI cohort** |  |  |  |  |
| TBI+ | 9% (9/98) | 10% (9/85) | 8% (6/81) | 9% (24/264) |
| TBI- | 24% (23/98) | 26% (22/85) | 26% (21/81) | 25% (66/264) |
| Sham | 11% (11/98) | 12% (10/85) | 12% (10/81) | 12% (31/264) |
| No phenotype | 56% (55/98) | 52% (44/85) | 54% (44/81) | 54% (143/264) |
|  |  |  |  |  |
| **EEG cohort** |  |  |  |  |
| TBI+ | 8% (7/86) | 6% (6/108) | 6% (4/66) | 7% (17/260) |
| TBI- | 42% (36/86) | 28% (31/108) | 20% (13/66) | 31% (80/260) |
| Sham | 16% (14/86) | 6% (6/108) | 11% (7/66) | 10% (27/260) |
| No phenotype | 34% (29/86) | 60% (65/108) | 63% (42/66) | 52% (136/260) |
|  |  |  |  |  |
| **Total (MRI and EEG)** |  |  |  |  |
| TBI+ | 9% (16/184) | 8% (15/193) | 7% (10/147) | 8% (41/524) |
| TBI- | 32% (59/184) | 28% (53/193) | 23% (34/147) | 28% (146/524) |
| Sham | 14% (25/184) | 8% (16/193) | 12% (17/147) | 11% (58/524) |
| No phenotype | 45% (84/184) | 56% (109/193) | 58% (86/147) | 53% (279/524) |

***Abbreviations:*** EEG, electroencephalography; MRI, magnetic resonance imaging; TBI+, rats with epilepsy; TBI-, rats without epilepsy.

**Supplementary Table 2.** Percentage of rats with electrode implantations.

|  | **UEF** | **Monash** | **UCLA** | **Total** |
| --- | --- | --- | --- | --- |
| **MRI cohort** |  |  |  |  |
| Excluded | 20 % (11/55) | 25 % (11/44) | 5 % (2/44) | 17 % (24/143) |
| Included | 100 % (43/43) | 100 % (41/41) | 100 % (37/37) | 100 % (121/121) |
| Total | 55 % (54/98) | 61 % (52/85) | 48 % (39/81) | 55 % (145/264) |
| **EEG cohort** |  |  |  |  |
| Excluded | 83 % (24/29) | 49 % (32/65) | 7 % (3/42) | 43 % (59/136) |
| Included | 100 % (57/57) | 100 % (43/43) | 100 % (24/24) | 99 % (123/124) |
| Total | 94 % (81/86) | 69 % (75/108) | 41 % (27/66) | 70 % (183/260) |
| **Total** |  |  |  |  |
| Excluded | 42 % (35/84) | 39 % (43/109) | 6 % (5/86) | 30 % (83/279) |
| Included | 100 % (100/100) | 100 % (84/84) | 100 % (61/61) | 100 % (245/245) |
| Total | 73 % (135/184) | 66 % (127/193) | 45 % (66/147) | 63 % (328/524) |

***Abbreviations:*** EEG, electroencephalography; MRI, magnetic resonance imaging; TBI+, rats with epilepsy; TBI-, rats without epilepsy.
